# The impact of three *SMN2* gene copies on clinical characteristics and effect of disease-modifying treatment in patients with spinal muscular atrophy: a systematic literature review

**DOI:** 10.3389/fneur.2024.1308296

**Published:** 2024-02-29

**Authors:** Claudia Dosi, Riccardo Masson

**Affiliations:** Fondazione IRCCS Istituto Neurologico Carlo Besta, Developmental Neurology Unit, Milan, Italy

**Keywords:** nusinersen, onasemnogene abeparvovec, prognosis, *SMN2* gene copy, spinal muscular atrophy

## Abstract

**Objective:**

To review the clinical characteristics and effect of treatment in patients with spinal muscular atrophy (SMA) and three copies of the *SMN2* gene.

**Methods:**

We conducted a literature search in October 2022 to identify English-language clinical research on SMA that included *SMN2* copy number according to PRISMA guidelines.

**Results:**

Our search identified 44 studies examining the impact of three *SMN2* copies on clinical characteristics (21 on phenotype, 13 on natural history, and 15 on functional status and other signs/symptoms). In children with type I SMA or presymptomatic infants with an *SMN1* deletion, three *SMN2* copies was associated with later symptom onset, slower decline in motor function and longer survival compared with two *SMN2* copies. In patients with SMA type II or III, three *SMN2* copies is associated with earlier symptom onset, loss of ambulation, and ventilator dependence compared with four *SMN2* copies. Eleven studies examined treatment effects with nusinersen (nine studies), onasemnogene abeparvovec (one study), and a range of treatments (one study) in patients with three *SMN2* copies. In presymptomatic infants, early treatment delayed the onset of symptoms and maintained motor function in those with three *SMN2* copies. The impact of copy number on treatment response in symptomatic patients is still unclear.

**Conclusion:**

*SMN2* copy number is strongly correlated with SMA phenotype in patients with *SMN1* deletion, while no correlation was found in patients with an *SMN1* mutation. Patients with three *SMN2* copies show a highly variable clinical phenotype. Early initiation of treatment is highly effective in presymptomatic patients with three *SMN2* copies.

## 1 Introduction

Spinal muscular atrophy (SMA) is a rare autosomal recessive condition, with an incidence of approximately 1:15,000 live births (1, 2) and greater prevalence in regions with high consanguinity rates (3). Affected individuals develop progressive muscle weakness and atrophy as a result of degeneration of motor neurons in the anterior horn of the spinal cord and brain stem nuclei (3). Muscle weakness is generally symmetrical and worse in proximal than distal muscle groups.

The severity of clinical presentation differs markedly between individuals, and may be classified into one of five clinical phenotypes depending on the maximal level of motor function achieved and age of onset (Supplementary Table S1), with type 0 being the worst (usually resulting in death shortly after birth) and type IV the mildest (onset in adulthood) (3). Almost all patients with SMA have a homozygous deletion of the *SMN1* gene, which codes for the survival motor neuron (SMN) protein (4). However, there are multiple other genetic factors that influence the phenotypic expression of SMA, including *SMN2* gene variants and how many copies of the *SMN2* gene the patient has (4).

A higher *SMN2* copy number is generally associated with a milder disease phenotype, but there is considerable variability in the clinical presentation of SMA within subgroups of patients based on *SMN2* copy number, and discordance between the expected phenotype based on *SMN2* copy number (5). Variability in clinical presentation is most marked in patients with three *SMN2* copies (6).

In the past 5–7 years, disease-modifying therapies have become available for the treatment of SMA, including nusinersen, risdiplam, and onasemnogene abeparvovec, which are affecting the expected disease course of SMA patients. These treatments are expensive (7), and healthcare payers may use selection criteria to reimburse treatment for those most likely to achieve benefit, including *SMN2* copy number (8). Such decisions are likely to be especially difficult in patients with three *SMN2* copies, who have a more variable clinical presentation. Therefore, the aim of the current systematic literature review is to comprehensively map the clinical characteristics of patients with three *SMN2* copies and to identify the effect of disease-modifying treatment in this group.

## 2 Materials and methods

We conducted a systematic literature review and report according to the Preferred Reporting Items for Systematic Reviews and Meta-Analyses (PRISMA) standards (9). A literature search was undertaken in October 2022 of the PubMed, Medline, Web of Science, and Cochrane databases using a combination of MeSH terms and free text items to identify articles on SMA that included *SMN2* copy number (see Supplementary Methods for full search strategies). No date limits were included. The search results were de-duplicated, and then articles were manually reviewed for inclusion based on the following criteria: (1) English language, and (2) clinical studies reporting *SMN2* copy number, which allows identification/definition of subgroups of patients with three *SMN2* copies.

Articles were excluded if they were published in languages other than English, or if they were animal or *in vitro* studies, conference presentations or proceedings, case reports or case series in which fewer than five patients had three or more *SMN2* copy numbers, review articles, articles about diseases other than SMA, studies focused on the methodologic detection of *SMN2* copies, or studies in which *SMN2* copy number was not reported.

From the remaining papers, the authors closely assessed the articles and chose those that specifically examined the impact of three *SMN2* copies on disease characteristics or treatment effect.

### 2.1 Bias assessment

Studies examining the relationship between *SMN2* copy and clinical parameters were assessed for bias using the Appraisal tool for Cross-sectional Studies (AXIS) (10), or the Newcastle-Ottawa Scale (NOS) for cohort or case–control studies (11). The AXIS tool poses 20 questions (one relating to the study introduction, 10 about methods, five about the presentation of the results, two about the discussion of study findings and limitations, one about funding, and one about ethics). Each question is answered with “yes,” “no,” or “do not know” (10). The AXIS tool does not provide an overall rating of bias. The NOS tools assess eight study design elements across three key domains in case–control and cohort studies (11). In case–control studies, the domains are selection of cases and controls (four elements – definition and representativeness of cases and controls), comparability of groups (one element – matching or controlling for confounders) and exposure (three elements – ascertainment of exposure and non-responder rate) (11). In cohort studies, the domains are similar: selection of cohort (four elements – definition and representativeness of cohort, selection of non-exposed cohort, and ascertainment of exposure), comparability of exposed and non-exposed groups (one element – matching or controlling for confounders), and outcome (three elements – ascertainment of outcome, follow-up duration and adequacy of cohort follow-up). Assessors of bias using NOS can apply star ratings of between one and nine stars for each study, with 0–4 stars for selection, 1 or 2 for comparability, and 0–3 for exposure (case–control) or outcomes (cohort) (11). We took account of the rarity of SMA and disease characteristics when applying the NOS. For example, for the assessment of outcomes, NOS gives one star for blinded assessment, but we gave the study one star if outcomes were assessed by trained evaluators using well-established rating scales, even if assessors were not blinded, because blinding is likely not possible in SMA cohort or case–control studies.

Non-randomized studies assessing the impact of *SMN2* copy number on treatment outcomes were assessed for bias using the Risk Of Bias In Non-randomized Studies—of Interventions (ROBINS-I) tool (12). This tool is designed to examine how much the study deviates from the ‘gold standard’ randomized, double-blind study. Seven potential sources of bias (domains) are assessed, and each is graded as having a low, moderate, serious, or critical risk of bias. These domains are: (1) confounding; (2) selection of participants into the study; (3) classification of interventions; (4) deviations from intended interventions; (5) missing data; (6) measurement of outcomes; and (7) selection of reported results (12). From these assessments, an overall risk of bias is estimated as low if the study is at low risk of bias in all domains, moderate if the study is at low or moderate risk of risk in all domains, serious if there is a serious risk of bias in at least one domain, and critical if there is a critical risk of bias in at least one domain. Studies with a critical risk of bias were not included in the current analysis, as these studies are considered too problematic to provide any useful evidence (12).

Randomized studies were assessed for bias using the revised Cochrane Risk-of-Bias tool for randomized trials (RoB 2) (13). This tool ranks five potential sources of bias (domains) as having low risk of bias, some concerns, or high risk of bias. The domains are: (1) bias arising from the randomization process; (2) bias due to deviations from the intended interventions; (3) bias due to missing outcome data; (4) bias in the measurement of the outcome; and (5) bias in the selection of the reported results. The overall risk is based on the following criteria: low if the study is judged to be at low risk of bias for all domains; some concerns if there are some concerns in at least one domain; and high if the study has a high risk of bias in at least one domain (14).

Data extraction and bias assessments were undertaken by CR and reviewed/confirmed by CD and RM.

## 3 Results

### 3.1 Literature search results and included studies

**Figure 1:**
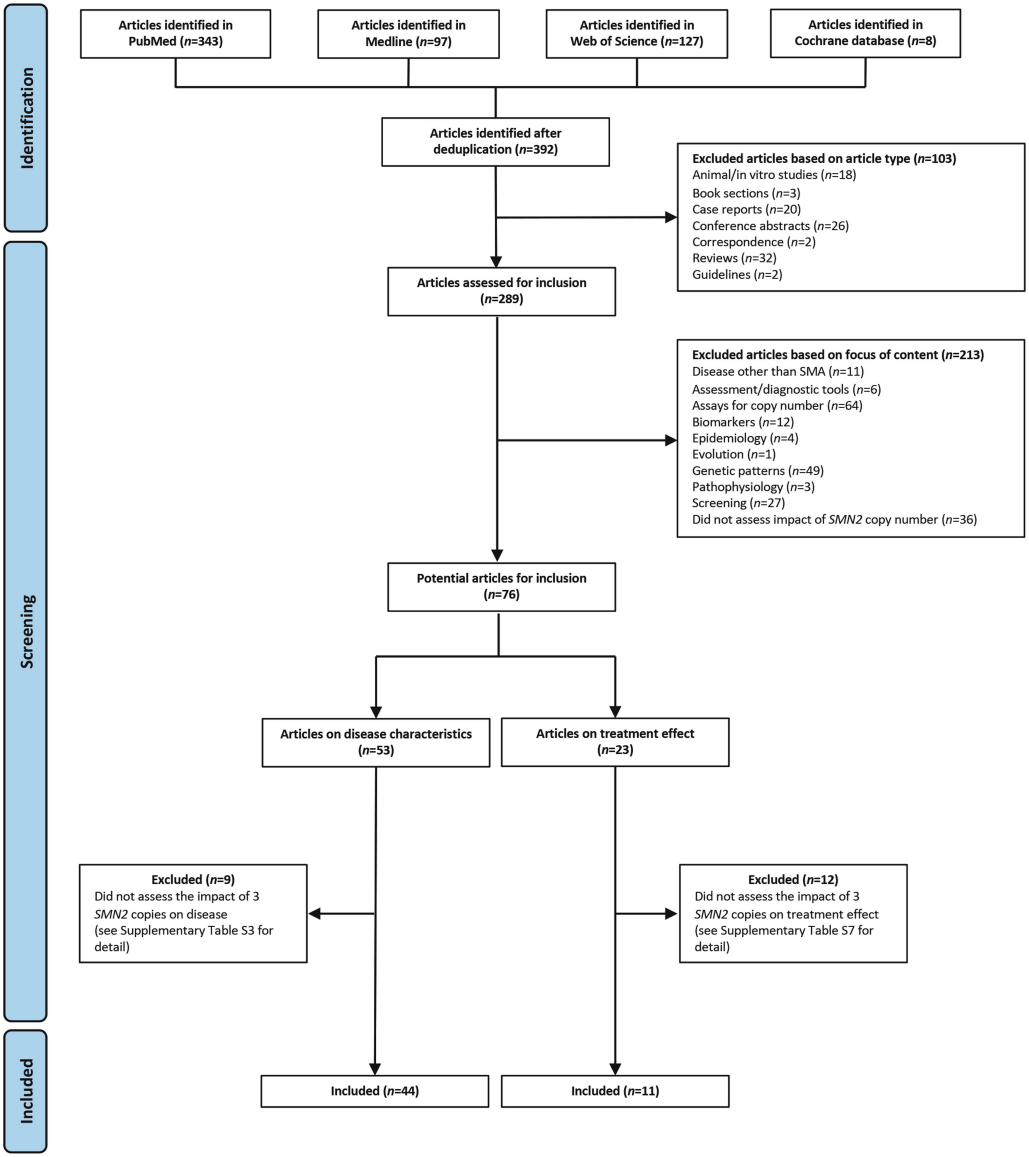
Search results and inclusion of studies. SMA, spinal muscular atrophy; SMN, survival motor neuron.

The search identified 392 studies. Of these, 103 were excluded because of the article type and 213 were excluded because of the focus of the content (Figure 1). Overall, 76 articles were reviewed for inclusion, of which 53 assessed disease characteristics and 23 assessed treatment effect.

### 3.2 Impact of three *SMN2* copies on disease characteristics

#### 3.2.1 Search results

Of the 53 articles assessing disease characteristics, 44 described the impact of three *SMN2* copies and are included in this review (Supplementary Table S2) (6, 15–57); nine articles were excluded because they did not assess the clinical impact of having three *SMN2* copies (Supplementary Table S3) (58–66). Of the 44 included studies on disease characteristics, 25 were prospective, 13 were retrospective, two were case–control studies, one was a post-hoc analysis of a cohort of patients from a randomized controlled trial (RCT), and three provided insufficient information on study design to make a determination (Supplementary Table S2). Where the site of the study could be determined (43 studies), two were multinational studies, five were conducted in Italy, five in Japan, three in China, and three in the USA (Supplementary Table S2).

Of the 44 studies, 43 reported SMA types. Overall, 23 included patients with a mix of SMA phenotypes ranging from type 0/I to type III or IV; the others included type 0/I only (six studies), type I or II only (three studies), type II only (two studies), type II or III only (two studies), type II to IV (two studies), type III only (three studies), type III or IV (one study), and one study compared type I and type III.

#### 3.2.2 Risk of bias

The cross-sectional studies assessing the relationship between *SMN2* copy number and clinical features were all consistent in not including a justification of sample size, and most studies did not discuss limitations (Supplementary Table S4). The studies were generally consistent in a number of features assessed by the AXIS tool, specifically clear study aims, an appropriate design, definition of the sample population, complete presentation of results, complete funding information/no evidence of potential conflicts of interest, and description of ethics (Supplementary Table S4). None of the studies in this analysis included a non-responder group, so three AXIS domains were consistently not relevant (questions 7, 13, and 14). Variability between studies was seen in AXIS domains related to the reporting of study population representativeness (questions 5 and 6).

Most studies scored highly using the NOS cohort or case–control tool, obtaining overall ratings of five (15, 21, 50, 57), six (26, 28, 29, 43, 54), or seven (32, 34, 42, 44, 45, 48, 51–53, 55) stars (Supplementary Table S5). One study obtained four stars (17); bias may be present in this study because there was limited description of how the study population was derived and 25% of patients were not followed up. The major sources of bias in the cohort and case–control studies were the proportion of patients lost to follow-up and the variability and/or adequacy of follow-up duration (Supplementary Table S5).

#### 3.2.3 SMA phenotype

Of the 44 studies, 21 assessed the relationship between *SMN2* copy number and SMA phenotype, and 19/21 studies reported the number of patients with three *SMN2* copies within each phenotype group (Table 1) (6, 15, 16, 18, 19, 22, 24, 30, 33, 35, 36, 38, 40–42, 46, 50, 52, 55).

**Table 1:** Studies evaluating the impact of three *SMN2* copies on clinical phenotype.

Author, yr.	No. (%) with 3 *SMN2* copies				No. (%) with ≥3 *SMN2* copies				Statistical analysis
	I	II	III	IV	I	II	III	IV	
Harada et al. 2002 (15)	3/11 (27.3)	14/14 (100.0)	1/2 (50.0)	0	3/11 (27.3)	14/14 (100.0)	2/2 (100.0)	–	S (correlation)
Mailman et al. 2004 (16)	2/52 (3.9)	0	70/90 (77.8)	0	2/52 (3.9)	0	90/90 (100.0)	0	S (difference)
Cuscó et al. 2006 (18)	2/16 (12.5)	8/14 (57.1)	9/15 (60.0)	0	2/16 (12.5)	8/14 (57.1)	13/15 (86.7)	0	NR
Wirth et al. 2006 (19)	0	0	IIIa: 30/60 (50.0)	0	0	0	IIIa: 53/60 (88.3)	4/4 (100.0)	S (correlation)^a^
			IIIb: 16/51 (31.4)				IIIb: 49/51 (96.0)		
Arkblad et al. 2009 (22)	0	10/11 (90.9)	4/14 (28.6)	0	0	11/11 (100.0)	14/14 (100.0)	0	S (correlation)
Watihayati et al. 2009 (24)	0	16/28 (57.0)		0	0	20/28 (71.4)		0	NR
Sifi et al. 2013 (30)	4/15 (26.7)	7/12 (58.3)	5/33 (15.2)	0	4/15 (26.7)	10/12 (83.3)	33/33 (100.0)	2/2 (100.0)	NR
Yamamoto et al. 2014 (33)	10/48 (20.8)	34/35 (97.1)	13/19 (68.4)	1/4 (25.0)	10/48 (20.8)	34/35 (97.1)	18/19 (94.7)	4/4(100.0)	S (difference)
Brkušanin et al. 2015 (35)	2/23 (8.7)	37/37 (100.0)	15/39 (38.5)	0	2/23 (8.7)	37/37 (100.0)	39/39 (100.0)	0	S (correlation)
Qu et al. 2015 (36)	40/106 (37.7)	97/101 (96.0)	16/25 (64.0)	0	41/106 (38.7)	100/101 (99.0)	25/25 (100.0)	0	S (correlation)
Medrano et al. 2016 (38)	0/4 (0.0)	4/4 (100.0)	4/6 (66.7)	0	0/4 (0.0)	4/4 (100.0)	6/6 (100.0)	0	NR
Kaneko et al. 2017 (40)	Exon 7: 5/24 (20.8)	Exon 7: 15/31 (48.4)	Exon 7: 5/11 (45.5)	0	Exon 7: 5/24 (20.8)	Exon 7: 17/31 (54.8)	Exon 7: 10/11 (90.9)	0	NS (difference)
	Exon 8: 5/24 (20.8)	Exon 8: 21/31 (67.7)	Exon 8: 5/11 (45.5)		Exon 8: 5/24 (20.8)	Exon 8: 23/31 (74.2)	Exon 8: 10/11 (90.9)		
Calucho et al. 2018 (41)	17/272 (6.3)	162/186 (87.1)	107/167 (64.1)	0	17/272 (6.3)	162/186 (87.1)	160/167 (95.8)	0	NR
De Sanctis et al. 2018 (42)	4/15 (26.7)	0	0	0	4/15 (26.7)	0	0	0	NR
Hryshchenko et al. 2020 (46)	7/59 (11.9)	10/49 (20.4)	10/34 (29.4)	0	8/59 (13.6)	11/49 (22.4)	22/34 (64.7)	0	S (difference)^b^
Wadman et al. 2020 (6)	29/59 (49.2)	109/120 (90.8)	27/98 (27.6)	0	30/59 (50.8)	118/120 (98.3)	95/98 (96.9)	9/9 (100.0)	S (correlation)
Lusakowska et al. 2021 (50)	80/140 (57.1)	149/182 (81.9)	185/344 (53.8)	1/6 (16.7)	85/140 (60.7)	167/182 (91.8)	323/344 (93.9)	6/6 (100.0)	NR
Wijaya et al. 2021 (52)	4/10 (40.0)	0	0	0	4/10 (40.0)	0	0	0	NR
Maggi et al. 2022 (55)	0	13/21 (61.9)	42/141 (29.8)	1/3 (33.3)	0	NA^c^	NA^c^	2/3 (66.7)	S (difference)

^a^Significant correlation between ≥4 *SMN2* copies with type IIIb vs. ≤3 copies with type IIIa.^b^Significantly higher proportion of type III patients (66.7%) had 3–5 *SMN2* copies compared with types 0–II (*p* < 0.0001).^c^Could not be calculated because only the number of patients with 2, 3, or 4 *SMN2* copies were reported, and it seems that some patients with SMA type II or III had 1 or ≥5 *SMN2* copies, based on the total N-values in those groups.NA, not available; NR, not reported; NS, not significant; S, statistically significant; SMN, survival motor neuron; yr., year.

Wijaya and colleagues (52) reported results that were somewhat anomalous to the usual pattern of milder phenotype with increasing *SMN2* copy number. This study examined only SMA patients with an intragenic variant of *SMN1* (excluding those with *SMN1* deletion), and found that 4/10 patients with SMA type I, but none of those with SMA type II or III, had three copies of *SMN2* (52).

A study by De Sanctis and colleagues included only patients with SMA type I, and reported that 4/15 infants had three *SMN2* copies; three with a milder phenotype and late onset and one with a typical type I phenotype (42).

A statistically significant effect of *SMN2* copy number on SMA phenotype was reported in all but one of the 12 studies that undertook statistical analysis (6, 15, 16, 19, 22, 33, 35–37, 40, 46, 55). Excluding the study by Wijaya and colleagues (52), the studies reported that three *SMN2* copies were present in between 0 and 57.1% of patients with SMA type I, 20.4%–100.0% of patients with SMA type II, 15.2%–77.8% of those with SMA type III, and 0%–33.3% of those with SMA type IV (Table 1). While the proportion of type II SMA patients with three or more *SMN2* copies was quite variable (22.4%–100.0%), 65%–100% of patients with type III or type IV SMA had three or more *SMN2* copies (Table 1).

Two other studies examined the relationship between SMA phenotype and *SMN2* copy number but did not report the number of patients with three *SMN2* copies in different phenotype groups (37, 47). Mendonça and colleagues compared patients who were homozygous for an *SMN1* deletion with those who were compound heterozygotes (47). They reported that, in homozygous SMA patients, 65% of those with SMA type II and 69.6% of those with SMA type III had three *SMN2* copies, and there was a clear relationship between phenotype and *SMN2* copy number. However, no correlation was found between *SMN2* copy number and disease phenotype in patients with a compound heterozygous genotype (47). This is consistent with the findings by Wijaya and colleagues in patients with *SMN1* intragenic variants described above (52).

Zarkov and colleagues reported the mean number of *SMN2* copies in patients with SMA type II, III, or IV, and found that the mean number of copies was higher in milder phenotypes (mean 3.1 in type II, 3.7 in type III and 4.2 in type IV; *p* < 0.05) (37).

Several studies, including the one by De Sanctis and colleagues described above, noted discordance between *SMN2* copy number and expected clinical phenotype. Lusakowska and colleagues reported that 85/140 patients with type I SMA (60.7%) in the Polish SMA registry had three or more *SMN2* copies (50); the cohort of 285 Dutch patients reported by Wadman and colleagues contained 30 patients with type I SMA who had three or more copies of *SMN2* (6); and Hryshchenko and colleagues found three *SMN2* copies in two patients with the most severe form of SMA (type 0) (46). In fact, Wadman and colleagues noted that phenotype was most variable among patients with three *SMN2* copies, ranging from those unable to sit independently (type Ic) to ambulant patients (type IIIa) (6).

#### 3.2.4 Natural history (age at onset and prognosis)

Of the 44 studies, 13 reported on the impact of *SMN2* copy number on clinical course/prognosis (17, 21, 23, 32, 36, 37, 39, 42, 49–51, 55, 57), of which eight provided data on age at onset and/or mortality (Table 2) (21, 23, 36, 49–51, 55, 57).

**Table 2:** Studies examining the relationship between *SMN2* copy number and natural history.

Author, yr	Study design	SMA type	No. with 3 *SMN2* copies/Total n	Patients with 3 *SMN2* copies			
				Age at onset	Age at death or last follow-up	Survival time	2-year survival probability
**Patients with type I SMA**							
Cobben et al. 2008 (21)	Prospective, longitudinal	I (*n* = 34)	3/34	Mean 55 days	Mean 690 days^a^	NR	NR
Rudnik-Schӧneborn et al. 2009 (57)	Retrospective	I (*n* = 66)	5/66	2–4.5 (mean 3.5) mos	10–55 (mean 31.4) mos^b^	NR	67%^c^
Ou et al. 2021 (51)	Retrospective	I (*n* = 111)	12/111	0–6 (mean 1.2) mos	NR	2.2–259.5 (median 60.7) mos	>60% ^d^
**Patients with type II or III SMA**							
Elsheikh et al. 2009 (23)	*Post hoc* analysis of RCT population	II or III	29/45	4.4 yrs	NR	NR	NR
**Patients with type III SMA**							
Lusakowska et al. 2021 (50)	Prospective	III (*n* = 232)	133/232	1 months to 18 yrs. (mean 3.01 yrs)	NR	NR	NR
**Patients with a mix of SMA types**							
Qu et al. 2015 (36)	Prospective, observational	I (*n* = 106), II (*n* = 101), III (*n* = 25)	153/232	0–27 (median 7) mos	NR	Mean 184 mos	97.4
Zhang et al. 2020 (49)	Prospective, cohort	I (*n* = 13), II (*n* = 16), III (*n* = 11)	25/40	Mean 17.8 mos	Mean 10.5 mos ^e^	NR	NR
Maggi et al. 2022 (55)	Retrospective, cross-sectional	II (*n* = 21), III (*n* = 141), IV (*n* = 3)	56/165	0.5–23 (mean 4.61) yrs	NR	NR	NR

^a^Includes 2 patients alive at the end of the study period.^b^All patients were alive at the end of the study period.^c^Estimated probability of surviving >2 yrs.^d^Exact figure not reported; this is an estimate from the Kaplan–Meier curve in the article by Ou et al. (51).^e^Mean age of the 2 patients who died. mo(s), month(s); NR, not reported; RCT, randomized controlled trial; SMN, survival motor neuron; yr(s), year(s).

Three studies reported the age at onset in children with type I SMA and three *SMN2* copies; the mean age was 1–2 months in two studies (21, 51), and 3.5 months in the other (57). One study reported a similar age at onset in children with two or three *SMN2* copies (51), but the other two studies reported a later age at onset in type I SMA patients with three *SMN2* copies than in those with up to two *SMN2* copies (21, 57).

Prognosis tended to be better in type I patients with three vs. two *SMN2* copies in two studies (51, 57), but there were too few patients with three *SMN2* copies in the study by Cobben and colleagues to draw any conclusions about the relationship between survival and *SMN2* copy number (21). Survival was longer in type I SMA patients with three vs. two *SMN2* copies (median 60.7 vs. 9.2 months) in a Taiwanese cohort study, but the survival probability between the groups did not reach statistical significance (*p* = 0.0687) (51). Two of the studies in type I SMA patients reported the estimated 2-year probability of death in those with three *SMN2* copies, and the survival was 60%–67% in both studies (51, 57).

A small Italian study in patients with type I SMA (15 had *SMN2* copy number available) found consistently more rapid deterioration in motor function in patients with two *SMN2* copies than in those with three *SMN2* copies (42), although motor function decline was variable.

In patients with type II or III SMA, the mean age at clinical onset was significantly lower in patients with three vs. four *SMN2* copies [mean 4.4 vs. 7.5 years in one study (23) and mean 3.01 vs. 6.71 years in the other (50)].

Unsurprisingly, the studies in patients with a range of SMA phenotypes (I–IV) reported a wider range of ages at onset (Table 2) (36, 49, 55). Qu and colleagues reported that Chinese patients with three *SMN2* copies had a median age of onset of 7 months, compared with 1.15 months in those with two *SMN2* copies and 18 months in those with four *SMN2* copies; age at onset was strongly correlated with *SMN2* copy number (*r* = 0.72, *p* < 0.0001) (36). These researchers also found a significant association between *SMN2* copy number and mortality; patients with two *SMN2* copies had a much higher risk of mortality compared with those who had three or four *SMN2* copies [odds ratio (OR) 186; 95% confidence interval (CI) 91.1–1812.7; *p* < 0.0001] (36). Similar results were reported by Zhang and colleagues in their study of 40 Chinese patients with SMA type I (*n* = 13), II (*n* = 16), or III (*n* = 11) (49): mean age at onset was 6.1, 17.8, and 31.4 months, respectively, in those with two, three, and four *SMN2* copies. None of the five patients with four *SMN2* copies had died, compared with 2/25 with three *SMN2* copies (8.0%) and 5/10 (50.0%) with two *SMN2* copies; mean age at death was 10.5 months in the two patients with three *SMN2* copies vs. 5.2 months in the five patients with two *SMN2* copies (49).

In contrast to the two Chinese studies described above (36, 49), a retrospective cross-sectional study from Italy including patients with SMA II–IV reported a similar age at onset in patients with two or three *SMN2* copies (median of 2 years in both groups; mean of 7.47 years in those with two copies vs. 4.61 years in those with three copies), although it should be noted that only seven patients in this study had two *SMN2* copies whereas 54 had three copies and 74 had four copies (55).

A US study examined the impact of *SMN2* copy number on age at death or at the combined endpoint of death and requirement for ventilation for ≥16 h/day in 79 patients with SMA type I or II (32). Among those with SMA type I, the median age at the combined endpoint was 10.5 months for those with two *SMN2* copies but not reached for those with three *SMN2* copies (25th percentile age in this group was 22 months) (32).

**Figure 2:**
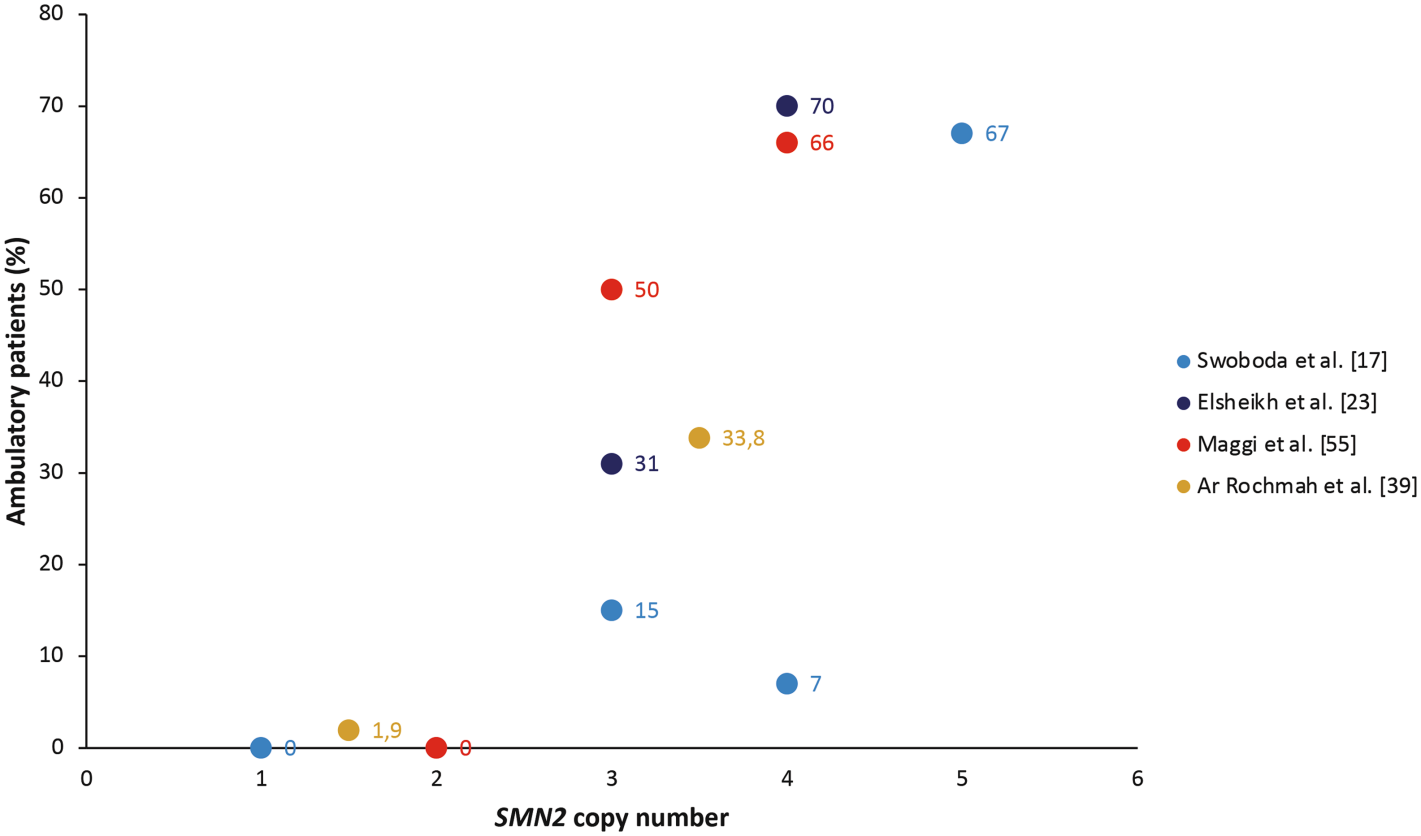
The proportion of ambulatory patients in cohort studies, stratified by *SMN2* copy number (17, 23, 39, 55). The study by Ar Rochmah et al. grouped patients into those with one or two *SMN2* copies compared with three or four *SMN2* copies (39).

Six studies noted a relationship between *SMN2* copy number and loss/retention of ambulation (17, 23, 37, 39, 50, 55). These populations also included patients with SMA II, who by definition never achieve ambulation, but generally showed a linear relationship between *SMN2* copy number and proportion of ambulatory patients in cohort studies (Figure 2). In their cohort of patients with SMA type II, III, or IV, Zarkov and colleagues noted that patients with two *SMN2* copies lost ambulation earlier than patients with three *SMN2* copies (after a mean of 23.7 vs. 37.4 years, respectively; *p* < 0.05), but found no significant correlation between *SMN2* copy number and achievement of motor milestones (37). *SMN2* copy number was also significantly associated with the probability of remaining ambulant over time in patients with type III SMA, with 70% of those with three *SMN2* copies still walking after 10 years and 60% after 20 years, compared with 91 and 82%, respectively, of those with four *SMN2* copies (*p* < 0.0001) in the study by Lusakowska and colleagues (50). Similarly, *SMN2* copy predicted the age at loss of ambulation in an Italian cohort of patients with SMA type II, III, or IV, although ambulation was lost at a younger age in this cohort than in the study by Zarkov and colleagues (12 years in those with two *SMN2* copies and 16.5 years in those with three *SMN2* copies) (55).

#### 3.2.5 Functional parameters and other signs/symptoms

Of 44 studies, 16 reported on the relationship between *SMN2* copy number and muscle strength, functional status (motor, neurologic, or pulmonary), and other signs/symptoms (Table 3) (17, 20, 23, 26, 28, 29, 31, 37, 42, 44, 45, 48, 53–56).

**Table 3:** Studies evaluating the impact of three *SMN2* copies on functional parameters.

Author, yr	SMA type	No. with 3 *SMN2* copies/Total *n*	Parameter	Key findings
**Muscle strength/electrophysiology**				
Swoboda et al. 2005 (17)	I (*n* = 26), II (*n* = 43), III (*n* = 14)	41/81	MUNE, CMAP	Greater deterioration in MUNE and CMAP over time in patients with ≤2 copies vs. ≥3 copies
Elsheikh et al. 2009 (23)	NR	29/45	MVIC	No difference in MVIC between patients with 3 vs. 4 copies
				Significant correlation between MVIC and copy number (correlation coefficient 0.63; *p* < 0.0001)
Kaufmann et al. 2011 (26)	II (*n* = 35), III (*n* = 30)	51/65	Myometry	No significant association between *SMN2* copy number and muscle strength deterioration
Farrar et al. 2013 (29)	I (*n* = 20), II (*n* = 31), IIIa (*n* = 14), IIIb (*n* = 5)	NR	CMAP	Significant correlation between CMAP and *SMN2* copy number (r-0.4; *p* = 0.05)
Tiziano et al. 2013 (31)	IIIa (*n* = 15), IIIb (*n* = 30)	13/45	Manual testing (MRC rating scale) and MVIC	No significant relationship between *SMN2* copy number and muscle strength
**Motor or neurologic function**				
Tiziano et al. 2007 (20)	II (*n* = 87)	46/87	HFMS	HFMS scores significantly lower in patients with 2 vs. 3 *SMN2* copies, after adjustment for age (*p* = 0.006)
Kaufmann et al. 2011 (26)	II (*n* = 35), III (*n* = 30)	51/65	GMFM, HFMSE	No significant association between *SMN2* copy number and functional change
Kaufmann et al. 2012 (28)	II (*n* = 41), III (*n* = 38)	61/79	GMFM, HFMSE	Significantly lower HFMSE and GMFM scores in patients with 3 *SMN2* copies vs. 4 copies
				No significant association between *SMN2* copy number and functional change over time
Farrar et al. 2013 (29)	I (*n* = 20), II (*n* = 31), IIIa (*n* = 14), IIIb (*n* = 5)	NR	HFMSE	Significant correlation between HFMSE and *SMN2* copy number in cross-sectional assessment (*r* = 0.5; *p* < 0.05)
				HFMSE showed an age-dependent decline over time in patients with 2 *SMN2* copies but remained relatively stable in patients with 3 copies
De Sanctis et al. 2018 (42)	I (*n* = 20)	3/15^a^	CHOP INTEND	More rapid decline in patients with 2 vs. 3 *SMN2* copies (statistical significance not reported)
Coratti et al. 2020 (44)	II (*n* = 243)	175/243	HFMSE	No significant association between *SMN2* copy number and gain/loss of functional ability
Coratti et al. 2020 (45)	IIIa (*n* = 136) or IIIb (*n* = 46)	78/182	HFMSE	No significant association between *SMN2* copy number and change in HFMSE score over time
Maggi et al. 2022 (55)	II (*n* = 21), III (*n* = 141), IV (*n* = 3)	56/165	HFMSE, RULM	No conclusions drawn about relationship between *SMN2* copy number and functional scores
Pane et al. 2022 (56)	0 (*n* = 1), I (*n* = 5), NC (*n* = 11)	3/17	HNNE	Significantly more patients with ≥3 vs. ≤2 *SMN2* copies had optimal HNNE scores
**Ambulatory function and standing**				
Tiziano et al. 2013 (31)	IIIa (*n* = 15), IIIb (*n* = 30)	13/45	NSAA, 6MWT	No significant relationship between *SMN2* copy number and MSAA or 6MWT
Townsend et al. 2020 (48)	I (*n* = 152) or II (*n* = 209)	163/361	Consistent stander use	*SMN2* copy number significantly predicted consistent stander use; children with 3 *SMN2* copies were 8 times more likely to consistently use a stander than patients with 2 copies (*p* < 0.001)
Krosschell et al. 2022 (54)	IIIa (*n* = 44), IIIb (*n* = 11), NC (*n* = 1)	25/56	10MWT	10MWT was 45% longer in patients with 3 vs. 4 *SMN2* copies (*p* = 0.013)
**Other signs/symptoms**				
Zarkov et al. 2015 (37)	II (*n* = 11), III (*n* = 17), IV (*n* = 8)	20/36	Limb contractures, spine deformities	No significant correlation between *SMN2* copy number and spine deformities or limb contractures
Hanna et al. 2022 (53)	I (*n* = 33), II (*n* = 39)	19/45	Hip pain	Hip pain present in 17% of patients with 2 *SMN2* copies vs. 53% with 3 *SMN2* copies and 50% with SMN4 copies (no statistical analysis of this relationship undertaken)
**Pulmonary function**				
Kaufmann et al. 2011 (26)	II (*n* = 35), III (*n* = 30)	51/65	FVC	No significant association between *SMN2* copy number and rate of change in FVC over time
Kaufmann et al. 2012 (28)	II (*n* = 41), III (*n* = 38)	61/79	FVC	No significant association between *SMN2* copy number and rate of change in FVC over time
Tiziano et al. 2013 (31)	IIIa (*n* = 15), IIIb (*n* = 30)	13/45	FVC	No significant association between *SMN2* copy number and FVC
Maggi et al. 2022 (55)	II (*n* = 21), III (*n* = 141), IV (*n* = 3)	56/165	FVC	Significantly lower FVC in patients with 3 vs. 4 *SMN2* copies (mean 74.49 vs. 92.5% predicted; *p* = 0.018 after adjustment)

*^a^SMN2* copy number were available for 15/20 patients.6MWT, 6-min walk test; 10MWT, 10-min walk test; CHOP INTEND, Children’s Hospital of Philadelphia Infant Test of Neuromuscular Disorders; CMAP, compound muscle action potential; FVC, forced vital capacity; GMFM, Gross Motor Function Measure; HFMS(E), Hammersmith Functional Motor Scale (Expanded); HNNE, Hammersmith Neonatal and Infant Neurological Examination; MRC, Medical Research Council; MUNE, motor units innervating a distal muscle group; MVIC, maximum voluntary isometric contraction; NSAA, North Star Ambulatory Assessment; NC, not classified; NR, not reported; SMN, survival motor neuron; RCT, randomized controlled trial; yr., year.

##### 3.2.5.1 Muscle strength/electrophysiology

Five studies investigated the relationship between neurophysiologic parameters and *SMN2* copy number (17, 23, 26, 29, 31).

Swoboda and colleagues found a greater deterioration in motor unit number estimation (MUNE) and compound muscle action potential (CMAP) over time among SMA patients with up to two *SMN2* copies compared with three or more copies (17), and Farrar et al. reported a significant correlation between *SMN2* number and CMAP (29). These studies were in a mixed population of patients with type I, II, or III SMA; however, the two studies that used myometry in patients with type II or III SMA found no significant relationship between muscle strength and *SMN2* copy number (26, 31).

Elsheikh and colleagues found no difference in maximum voluntary isometric contraction (MVIC) between patients with three vs. four *SMN2* copies, but did find a significant correlation between MVIC and *SMN2* copy number (23).

##### 3.2.5.2 Motor or neurologic function

One study examined neurologic function in infants identified by newborn screening (*n* = 17), and found that a significantly higher proportion of those with three or more *SMN2* copies had optimal scores on the Hammersmith Neonatal Neurological Examination (HNNE) scale compared with infants who had up to two *SMN2* copies (66.7% vs. 14.3%; *p* = 0.036) (56).

Eight studies examined the impact of *SMN2* copy number on motor function using the Hammersmith Functional Motor Scale (HFMS) or HFMS Expanded (HFMSE; seven studies) (20, 26, 28, 29, 44, 45, 55), gross motor function measure (GMFM; two studies) (26, 28), revised version of the upper limb module (RULM; one study) (55), and Children’s Hospital of Philadelphia infant test of neuromuscular disorders (CHOP INTEND; one study in children with type I SMA) (42).

The only study in children with SMA type I reported a more rapid decline in CHOP INTEND scores in patients with two vs. three *SMN2* copies, but *SMN2* copy number data were available for only 15/20 patients (75%), and no statistical analysis was undertaken on these findings (42).

In cross-sectional analyses in patients with SMA type II or III, HFMSE scores were significantly lower in patients with two vs. three *SMN2* copies (20), or those with three vs. four *SMN2* copies (28), and there was a significant correlation between *SMN2* copy number and HFMSE scores in patients with types I, II, or III (29).

On the other hand, the results of longitudinal assessments appeared to show no strong association between *SMN2* copy number and changes in motor function. Kaufmann and colleagues found no significant association between *SMN2* copy number and change in HFMSE or GMFM over time (26, 28). Similarly, Coratti and colleagues reported no significant association between *SMN2* copy number and gain/loss of functional ability over time in patients with type II SMA (44), or between *SMN2* copy number and HFMSE scores over time in patients with type III SMA (45). Farrar and colleagues reported an age-dependent decline in HFMSE scores among patients with two *SMN2* copies, but relatively stable HFMSE scores in those with three copies (29).

##### 3.2.5.3 Ambulatory function and standing

Two studies examined ambulatory function in adult patients with type III SMA using the North Star Ambulatory Assessment (NSAA) and 6-min walk test (6MWT) (31), or the 10-min walk test (10MWT) (54).

Tiziano and colleagues found no relationship between NSAA or 6MWT and *SMN2* copy number (31), whereas Krosschell and colleagues reported a 45% slower 10MWT among patients with three vs. four copies (*p* = 0.013) (54), in line with expectations. According to Townsend and colleagues (48), children with three *SMN2* copies were eight times more likely to consistently use a stander than patients with two copies (*p* < 0.001).

##### 3.2.5.4 Other signs/symptoms

Two studies examined the relationship between other signs/symptoms and *SMN2* copy number (37, 53).

Zarkov and colleagues reported no significant correlation between *SMN2* copy number and spine deformities or limb contractures in patients with type II, III, or IV SMA (37). Hanna and colleagues reported a higher incidence of hip pain among patients with type I or II SMA and three *SMN2* copies (53%) compared with two *SMN2* copies (17%). Only two patients in this study had four *SMN2* copies and one-half of them had hip pain (53).

##### 3.2.5.5 Pulmonary function

Four articles reported the relationship between forced vital capacity (FVC) and *SMN2* copy number in patients with SMA type II or III (26, 28), type III (31), or type II, III or IV (55).

The largest of these studies (in 165 patients with type II, III, or IV) found a significantly lower FVC in patients with three vs. four *SMN2* copies (74.49% vs. 92.5% predicted; *p* = 0.018) (55). However, in a cross-sectional study in type III patients, there was no significant association between FVC and *SMN2* copy number (31), and in a prospective longitudinal study in patients with type II or III SMA, there was no significant association between *SMN2* and change in FVC over time (26, 28).

#### 3.2.6 Protein expression/biomarkers

Two studies examined the relationship between *SMN2* copy number and plastin 3 (PLS3) expression in patients with type I, II, or III SMA (25, 34), and one examined the impact of *SMN2* copy number on SMN protein levels in patients with a range of SMA types (I–IV), including nine presymptomatic patients (Table 4) (43).

**Table 4:** Studies evaluating the impact of three *SMN2* copies on biomarkers.

Author, yr	Study design	Country	SMA type	No. with 3 *SMN2* copies/Total *n*	Key findings
**PLS 3 expression**					
Stratigopoulos et al. 2010 (25)	Prospective observational	USA	I (*n* = 23), II (*n* = 37), III (*n* = 28)	59/78	Significant correlation between *SMN2* copy number and PLS3 expression in postpubertal females but not pre- or postpubertal males or in prepubertal females
Yanyan et al. 2014 (34)	Case–control	China	I (*n* = 19), II (*n* = 21), III (*n* = 25)	50/65	No difference in PLS3 expression in patients with 2, 3 or 4 *SMN2* copies, but in females with 3 *SMN2* copies, PLS3 expression was significantly higher in those with type II than type III SMA
**SMN protein levels**					
Alves et al. 2020 (43)	Retrospective	USA	I (*n* = 8), II (*n* = 21), III (*n* = 35), IV (*n* = 1), presymptomatic (*n* = 9)	37/74	Significantly higher SMN protein levels in patients with 3 vs. 2 *SMN2* copies after controlling for age (*p* < 0.0001)

PLS3, plastin 3; SMN, survival motor neuron; yr., year.

The two studies of PLS expression suggest that PLS expression may modify the SMA phenotype in an age- and/or sex-specific manner. Stratigopolous and colleagues found a significant correlation between *SMN2* copy number and PLS3 transcript levels in postpubertal females, but not in prepubertal females or in males in either age group (25). In postpubertal females, PLS3 expression was also significantly correlated with motor function (assessed using GMFM) (25). A Chinese case–control study found no significant difference in PLS3 expression between patients with two, three, or four *SMN2* copies (34). However, in the subgroup of female patients with three *SMN2* copies, PLS expression was significantly higher in those with SMA type II vs. type III (34).

A US study found that SMN protein levels in whole blood were significantly higher in patients with three vs. two *SMN2* copies (*p* < 0.0001), and between patients with type II, III, or IV SMA (*p* < 0.0001), after controlling for age (43).

### 3.3 Impact of three *SMN2* copies on treatment effects

#### 3.3.1 Search results

Overall, 23 studies on SMA treatment included *SMN2* copy number (67–89), but only 11 of these studies examined treatment effects in patients with three *SMN2* copies (Supplementary Table S6) (68, 69, 71, 74, 79, 80, 83, 85, 87–89). The 12 excluded studies did not allow analysis of treatment effects in patients with three *SMN2* copies (Supplementary Table S7) (67, 70, 71, 73, 75–78, 81, 82, 84, 86).

Among the 11 included studies, nine were in patients receiving nusinersen (68, 69, 71, 74, 80, 83, 85, 88, 89), one was in patients receiving onasemogene abeparvovec (87), and one was in patients receiving a range of treatments, including nusinersen, onasemnogene abeparvovec, and risdiplam (79). Study design was not clearly defined in one of the nusinersen studies (68); the study of patients receiving a mix of treatments was retrospective (79), but all other studies were prospective (69, 71, 74, 80, 83, 85, 87–89). One nusinersen study was a subanalysis of the phase 2 NURTURE study (74), one was the phase 3 randomized CHERISH study (89), and the study with onasemnogene abeparvovec was a subanalysis of the SPR1NT study (87). The other prospective studies were observational cohort studies.

#### 3.3.2 Risk of bias

The non-randomized studies in this analysis had an overall ROBINS-I rating of moderate (six studies) or serious (three studies; Supplementary Table S8). In all non-randomized studies, the risk of bias from participant selection and missing data was low (Supplementary Table S8). However, all non-randomized studies had at least a moderate risk of confounding, which resulted in none of the studies having a low overall risk of bias.

The randomized study (CHERISH) had a RoB2 overall rating of ‘some concerns’. There was a low risk of bias due to deviations from intended interventions, missing outcomes data, outcome measurement or selection of reported results (Supplementary Table S8). However, there were some concerns about bias due to randomization because nusinersen or control was administered by personnel who were aware of the group assignments.

#### 3.3.3 Nusinersen

The nine studies in patients receiving nusinersen included a total of 506 patients, of whom 230 had three *SMN2* copies. Where SMA type was specified, 68 patients were presymptomatic infants; the others had type I (*n* = 296), type II (*n* = 14), or type III (*n* = 2) SMA. The SMA type was not specified in the CHERISH study cohort (*n* = 126), but these patients were considered to have type II or III SMA based on the inclusion criteria (89).

Seven studies – one subanalysis of the phase 2 NURTURE study in presymptomatic infants (74), one real-world study in patients diagnosed by newborn screening (88), and five prospective cohort studies in patients with type I SMA (68, 69, 80, 83, 85)—compared outcomes in patients with two vs. three or more *SMN2* copies. Of these seven studies, four reported baseline and follow-up data in groups with two vs. three *SMN2* copies, and are summarized in Table 5. There was an improvement in functional scores (CHOP INTEND, HINE-2, and MFM 20) during nusinersen treatment in patients with two or three or more *SMN2* copies in five studies (69, 74, 80, 83, 85). Only one study statistically compared patients with two vs. three *SMN2* copies, and found no significant difference in functional scores (HINE-2, CHOP INTEND, MFM-20 or − 32) between groups (69). However, another observational study reported that *SMN2* copy number was a significant predictor of outcomes among nusinersen-treated patients with type I SMA (83).

**Table 5:** Studies examining the effects of nusinersen in presymptomatic or type I SMA patients with three vs. two *SMN2* copies.

	2 *SMN2* copies		3 *SMN2* copies	
**Presymptomatic infants (** **74****)**	**Baseline (*n* = 15)**	**Follow-up^a^ (*n* = 15)**	**Baseline (*n* = 10)**	**Follow-up^a^ (*n* = 10)**
**Motor milestones, *n* (median age)**				
Sitting without support	–	15 (7.9 mo)	–	10 (6.4 mo)
Walking with assistance	–	13 (16.1 mo)	–	10 (9.6 mo)
Walking alone	–	12 (20.4 mo)	–	10 (12.3 mo)
HINE-2 scores, mean	2.7	23.9	3.2	26.0
CHOP INTEND scores, mean	47.0	62.1	51.9	63.4
**Patients showing protocol-defined SMA symptoms, *n* (%)**				
Age 13 mos	0	10 (67)	0	2 (20)
Age 24 mos	0	7 (47)	0	0
**Type I patients aged > 7 mos (** **69****)**	**Baseline (*n* = 15)**	**6 mos (*n* = 15)**	**Baseline (*n* = 17)**	**6 mos (*n* = 17)**
HINE-2 score, median	1	3^*^	2	4^**^
CHOP INTEND score, median	30.5	36.5^*^	32	34.5^*^
MFM 20 score, median	25	32.35	24.67	30
Ventilatory support, *n* (%)^b^	9 (60.0)	13 (86.7)	7 (41.2)	9 (52.9)
Feeding support, *n* (%)^c^	4 (26.7)	6/15 (40.0)	5 (29.4)	5 (29.4)
**Type I patients aged 2 mos to 15.9 yrs. (** **83****)**	**Baseline (*n* = 61)**	**12 mos (*n* = 61)**	**Baseline (*n* = 19)**	**12 mos (*n* = 19)**
CHOP INTEND score, mean	11.98	17.10	28.42	36.47
HINE-2 score, mean	0.41	1.58	1.58	4.71
**Type I patients of any age (median 23 mos) (** **68****)**	**Baseline (*n* = 26)**	**14 mos (*n* = 26)**	**Baseline (*n* = 18)**	**14 mos (*n* = 18)**
Achieved sitting status, *n*	–	8 (30.7)	–	7 (38.9)

^a^Up to day 778.^b^Non-invasive or invasive ventilation.^c^Nasogastric tube or percutaneous endoscopic gastrostomy.^*^p < 0.05 vs. baseline; ^**^p ≤ 0.001 vs. baseline.CHOP INTEND, Children’s Hospital of Philadelphia Infant Test of Neuromuscular Disorders; HINE, Hammersmith Infant Neurological Examination; MFM 20, Motor Function Measure (20 item); mo(s), month(s); SMA, spinal muscular atrophy; SMN, survival motor neuron; yr., year.

A study by Aragon-Gawinska and colleagues found that *SMN2* copy number had no significant effect on whether children with type I SMA achieved sitting status during nusinersen treatment (68). However, this study had a serious risk of bias, because patients were assigned to groups for analysis based on sitting status achieved after treatment, and there were differences between these groups with regard to cointerventions (68).

A Polish study reported that SMA type I patients with three or more *SMN2* copies had higher CHOP INTEND scores than those with two copies (*p* = 0.013), and tended to show a greater improvement during treatment, although this did not reach statistical significance (80).

The NURTURE subanalysis in presymptomatic patients showed that patients with three *SMN2* copies receiving nusinersen were less likely to develop SMA symptoms, and more consistently achieved motor milestones and at a younger median age compared with the group with two *SMN2* copies (Table 5), although the between-group difference was not statistically tested (74). This study had a moderate risk of bias, but was one of the few non-randomized studies to have a low risk of bias in outcome measurement because it was part of a clinical trial.

A separate study in infants identified by newborn screening found that presymptomatic patients treated with nusinersen remained asymptomatic and achieved normal motor milestones during follow-up, and this was true of eight patients with two *SMN2* copies and six with three *SMN2* copies (88). In contrast, all untreated infants with two or three *SMN2* copies developed symptoms (88). However, the study authors did not adjust for confounding, so this study had a serious risk of bias.

The randomized phase 3 CHERISH study compared nusinersen and sham control in patients with type II SMA or milder (later onset SMA), and found that nusinersen was significantly more effective than control at improving HFMSE score (the primary endpoint; *p* < 0.001) (89). A significant improvement was seen in all subgroups of patients based on *SMN2* copy number, but most patients in this study (111/126; 88%) had three *SMN2* copies (89). While there are some concerns regarding the risk of bias associated with potential differences in the baseline characteristics between the nusinersen and control groups (lower mean HFMSE score at baseline, longer median disease duration and higher proportion of patients who could walk with support in the control group than the nusinersen group), the randomized controlled nature of the study means that results can be considered robust.

A small US study investigated nusinersen in patients with type I, II or III SMA, but focused on the feasibility of using an indwelling subcutaneous catheter to administer nusinersen in patients with complex spinal anatomy (71). The study reported an improvement in sum total force (an aggregate of 11 dynamometry maneuvers) after starting nusinersen in one patient aged 6 years with four *SMN2* copies and in three patients aged 5–10 years with three *SMN2* copies, but not in six patients with three *SMN2* copies aged 13–30 years (71). Risk of bias in this study was considered to be moderate.

#### 3.3.4 Onasemnogene abeparvovec

Strauss and colleagues reported a subgroup analysis from the phase 3 SPR1NT study in presymptomatic infants at risk of developing SMA who had three *SMN2* copies (*n* = 15) (87). They did not directly compare outcomes in treated patients with three vs. two *SMN2* copies, because the primary study endpoint was different between groups (independent sitting at any visit up to 18 months of age in those with two *SMN2* copies vs. independent standing within 24 months of age in those with three *SMN2* copies) (90). All 15 infants with three *SMN2* copies achieved the primary endpoint (independent standing for ≥3 s at any visit up to 24 months of age); the median time to this milestone was 377 days, and 14/15 (93%) achieved independent standing within the normal developmental window, compared with 24% in the Pediatric Neuromuscular Clinical Research (PNCR) natural history study (*p* < 0.0001). Overall, 14/15 infants (93%) were able to walk independently for ≥5 steps at any visit up to 24 months of age, with a median age of independent walking of 422.0 days, and 11/15 (73%) within the normal developmental window (87).

Similar primary endpoint results were seen in the patients with two *SMN2* copies, with 14/14 of these patients (100%) achieving independent sitting at any visit up to 18 months of age (90). However, while 93% of patients with three *SMN2* copies achieved independent standing within the World Health Organization (WHO) developmental window (87), this endpoint was achieved by 50% of patients with two *SMN2* copies (90), and the median time to independent walking was 422 days in those with three copies vs. 493 days in those with two copies (87, 90).

All 15 of the patients with three *SMN2* copies treated with onasemnogene abeparvovec were alive and free from permanent ventilation at the end of the study (87). Risk of bias in this study was considered to be moderate.

#### 3.3.5 Any treatment

Lee and colleagues retrospectively assessed treatment patterns and outcomes in infants identified through the newborn screening program implemented in New York state in 2018 (79). Of the 34 infants identified in the screening program, 32 received treatment, of whom 11 had three *SMN2* copies. Treatments received by screened infants were onasemnogene abeparvovec alone (*n* = 23), nusinersen alone (*n* = 1), risdiplam alone (*n* = 1), nusinersen as a bridge to onasemnogene abeparvovec (*n* = 5), or risdiplam after initial treatment with onasemnogene abeparvovec (*n* = 2).

Ten of the 11 patients with three *SMN2* copies had received onasemnogene abeparvovec alone and the other received nusinersen as a bridge to onasemnogene. The 11 infants with three *SMN2* copies were all asymptomatic at the time of treatment (age 11–94 [median 34] days) and remained asymptomatic at last follow-up (age at last follow-up 1.5–26 months), when they had met age-appropriate developmental milestones and had normal neurologic function. All seven children with three *SMN2* copies aged ≥ 12 months at follow-up were walking independently. None of the treated patients with three *SMN2* copies required ventilatory or feeding support (79). Risk of bias in this study was considered to be serious based on the potential for confounding.

## 4 Discussion

This systematic review confirms that *SMN2* copy number is strongly correlated with SMA phenotype in patients with *SMN1* deletion. It also confirms that patients with three copies of the *SMN2* gene have a more variable clinical presentation/phenotype than patients with up to two copies or four or more copies, and that patients with three *SMN2* copies may have SMA ranging from severe (type I) to mild (type IIIc) (91). These data support the contention that *SMN2* copy number is not sufficient to explain the variability of clinical presentation in patients with SMA. The largest analysis of data on this issue to date indicates that three *SMN2* copies are present in 20% of type I patients, 78% of type II patients, and 49% of type III patients (41), in line with the published data in our analysis (Table 1).

Our analysis found no relationship between *SMN2* copy number and phenotype in SMA patients who carry an *SMN1* mutation, although this was examined in only two studies in a small number of patients (47, 52). Newborn screening identifies only patients with *SMN1* deletion (92), so patients with *SMN1* mutation will not be identified until symptoms develop. The lack of relationship between *SMN2* copy number and phenotype in these patients with *SMN1* mutation makes it difficult to provide prognostic information to parents of these children.

In relation to life expectancy in SMA type I, three studies consistently reported better odds of survival and/or longer survival duration in type I SMA patients with three copies than in those with two copies (21, 51, 57). Another study reported that *SMN2* copy number was a significant protective factor for survival with early death in 4/4 patients with only one *SMN2* copy (mean survival 4 months) (66). The mean *SMN2* copy number was significantly higher in type I patients who survived >2 years (mean 2.89 copies) than in those who died before the age of 2 years (mean 2 copies) (66). The lack of information on the impact of *SMN2* copy on survival duration in patients with types II, III, or IV SMA is not surprising, given the life expectancy of these patients (93), and the need for large cohorts and long follow-up to develop accurate data.

In addition, the data indicate that patients with three *SMN2* copies have an age at onset and time to ventilator dependence or loss of ambulation that is intermediate between patients with up to two copies or four or more copies. Cross-sectional studies using tests of ambulatory function (e.g., 6MWT) did not find a significant difference between groups based on *SMN2* copy number, which is consistent with other reports that these parameters show too much interindividual variation and overlap between phenotype groups to provide valuable information on their own (94). This indicates that, within the same class of motor milestones (e.g., walking), the number of *SMN2* copies is not sufficient to define clinical classification, even if there is an overall correlation with prognosis.

Our findings indicate that the relationship between *SMN2* copy number and neurophysiologic parameters depends on what is measured; CMAP was significantly related to *SMN2* copy number (17, 29), but myometric measures were not (26, 31). A relationship between CMAP amplitude and *SMN2* copy number has even been detected in presymptomatic patients identified by newborn screening, and CMAP may be a sensitive measure of motor function impairment in infants before overt symptoms develop (95). Our analysis also suggests that motor function (measured using standard scales such as HFMSE or CHOP INTEND) was better in patients with three vs. two *SMN2* copies, at least in cross-sectional studies, but the evidence from longitudinal studies did not consistently show a slower decline in motor function among patients with three vs. two *SMN2* copies.

While these data provide additional information on the natural history of SMA which may assist in the counseling of patients/parents about what to expect, *SMN2* copy number is only one factor moderating the clinical severity/phenotype of SMA patients. Therefore, the identification of other biomarkers is needed to guide phenotypic or prognostic estimations. *NAIP* copy number also shows a relationship with the clinical severity of SMA (6, 24, 27, 36, 49). Two groups of Chinese researchers used combined genotype information from *SMN1-SMN2-NAIP* as a prognostic marker, and both noted that patients with a 0–3-1 genotype were significantly less likely to develop type I SMA and to have significantly better survival compared with patients harboring the 0–2-0 genotype (36, 49). Within groups of patients with two or three *SMN2* copies, the presence of *NAIP* copies modified the risk of survival and disease progression (36, 49), suggesting that more nuanced genotyping will become part of the SMA prognostic algorithm in future. Some of the other biomarkers being considered in SMA include PLS3 and/or coronin 1C expression, SMN protein levels in blood, microRNA, neurofilament proteins, creatine kinase or creatinine levels, and Tau levels in the CSF (43, 94, 96–98). We identified only three papers evaluating the relationship between *SMN2* copy number and PLS3 or SMN protein levels (25, 34, 43). The authors of the study on SMN protein levels in whole blood speculated that this biomarker could provide adjunctive information (in addition to *SMN2* copy number) about the likely phenotype of young patients with SMA and help to inform treatment decisions (43). Further research is needed to identify biomarkers relevant to the natural history and treatment response of patients with SMA.

Our review identified 11 studies examining the impact of *SMN2* copy number on treatment effects, but like previous systematic reviews (99), we did not identify any studies that assessed the impact of copy number on survival in treated patients. To date, the largest number of publications are about nusinersen. Based on the available data, nusinersen appears to be as effective in patients with three *SMN2* copies as in those with up to two copies (68, 69, 71, 74, 80, 83, 85, 88, 89). However, there is potential for bias in observational assessments, particularly when *SMN2* copy number is related to disease phenotype, and therefore to decisions about treatment. In the studies that presented baseline clinical characteristics in the groups with two vs. three *SMN2* copies, age-related motor function at baseline tended to be better and age at first nusinersen dose tended to be higher in those with three vs. two copies (69, 74). Even if age at first dose is comparable, the expected slower decline in motor function in untreated patients with three vs. two *SMN2* copies complicates the comparison of treatment response between copy number groups (17, 29, 42).

The randomized phase 3 CHERISH study was the only study to include a control group, and the data showed similar motor function improvement during nusinersen treatment in the patients with three vs. two *SMN2* copies; however, only 9% of patients in this analysis had two *SMN2* copies compared with 87% with three *SMN2* copies (89), so the data should be confirmed in larger studies.

The SPR1NT study examined the effect of *SMN2* copy number on gene therapy (onasemnogene abeparvovec) efficacy in presymptomatic patients, and found that outcomes were significantly better in treated patients with three *SMN2* copies than in a historical control group of untreated patients with two or three *SMN2* copies (87). However, the lack of a direct comparator hampered interpretation of these findings.

As newborn screening becomes more widespread, a growing proportion of the candidates for treatment are presymptomatic infants, and questions arise about the cost-effective application of treatment-modifying therapies, particularly in infants with a milder phenotype. The European *ad hoc* guidelines for the use of gene therapy in SMA recommend that, in presymptomatic infants, *SMN2* copy number should be used to select patients for treatment, because it is currently the most accurate predictor of age at onset and clinical severity (100). In all other patients, age at onset, disease duration, and motor function status are the most important factors that predict response to pharmacologic treatments.

Our review identified three studies on the use of nusinersen in this setting [the open-label phase 2 NURTURE study (74), a prospective cohort study (88), and a retrospective study (79)] and two on onasemnogene abeparvovec [a retrospective study (79) and the SPR1NT study (87, 90)], but no published studies on risdiplam. These data showed that early use of disease-modifying treatment delayed the onset of symptoms and maintained motor function in patients with three *SMN2* copies. A recent systematic review of clinical trials and real-world studies examining the impact of treatment in presymptomatic infants seems to confirm these findings, although a longer follow-up will be necessary to verify the clinical outcome of these patients (1). Real-world data show that almost all infants with three *SMN2* copies who began treatment with disease-modifying therapy within the first 6 weeks of life had normal motor development and reached age-appropriate milestones; infants with two *SMN2* copies also derive considerable benefit but the proportion of patients achieving normal motor development was smaller in this group than in those with three *SMN2* copies (1).

Quantification of *SMN2* copy number is not straightforward and a number of different techniques may be used (5). The most common technique is multiplex ligation-dependent probe amplification (MLPA), but results are not always concordant between MLPA assay kits or between laboratories using the same assays (88, 101). European guidelines recommend that *SMN2* copy number analysis is undertaken by an expert laboratory with effective quality control measures in place (100).

*SMN2* copy number may not align with expectations based on the patient phenotype (5), and our review suggests that this is most likely in patients with three *SMN2* copies. A number of specific mutations in *SMN2* may modify disease severity, and there is evidence of structural differences in the *SMN2* gene copies within the same patient (6). Patients with two or three *SMN2* copies and an unexpected phenotype may have rare positive variants of *SMN2* associated with a milder phenotype (e.g., c.859G>C or c.835-44A>G) (5, 6). However, if patients with one or four or more *SMN2* copies show a discordant phenotype, physicians should consider retesting for *SMN2* copy number with a new sample and/or a different laboratory or technique (5).

Because of the difficulties inherent in conducting clinical trials in patients with rare diseases (102), this systematic review included a small number of randomized trials, as only a few trials are conducted in patients with SMA. Our search identified only one RCT, so there was no opportunity for meta-analysis. However, this is to be expected in rare disease research where there are few patients and ethical concerns often preclude the use of placebo (102). Most studies were small, and many were excluded because they did not specifically assess the impact of three *SMN2* copies on outcomes. Differences in the study designs and endpoints limit the conclusions that could be drawn. Several pharmacologic studies have recently been presented at conferences, but were excluded from this analysis because the data have not been peer-reviewed.

Some studies in this review lacked sufficient detail in reporting study design to conduct a detailed assessment of bias. Moreover, almost all studies in our analysis were observational and potentially affected by bias. This may be particularly true in observational treatment studies where decisions around the use and timing of treatment are likely to be related to disease phenotype, which (as we have shown) is affected by *SMN2* copy number. For example, we used the ROBINS-I tool for the assessment of bias in non-randomized intervention studies (12); most non-randomized trials will be assessed as having at least moderate risk of bias using this tool (103).

## 5 Conclusion

This review of the available literature indicates that SMA patients with three *SMN2* copies show a more variable clinical presentation than those with one, two, four, or five copies. In infants and children with type I SMA or in presymptomatic infants with an *SMN1* deletion, having three *SMN2* copies is associated with a later onset of symptoms, a slower decline in motor function and longer survival compared with having two copies of *SMN2*. In patients with SMA type II or III, having three *SMN2* copies is associated with earlier symptom onset, loss of ambulation and ventilator dependence compared with having four *SMN2* copies. Early disease-modifying treatment (with nusinersen or onasemnogene abeparvovec) delays the onset of symptoms in presymptomatic patients, and may help these patients with three *SMN2* copies to remain asymptomatic and meet normal motor milestones, but the studies were mostly small and uncontrolled. In the RCT (CHERISH), nusinersen was as effective at improving motor function in patients with three *SMN2* copies as it was in those with two *SMN2* copies. Given the variable clinical phenotype of SMA patients with three *SMN2* copies, more research is needed in additional biomarkers to help the prognosis and response to treatment of patients with three *SMN2* copies.

## Data availability statement

The original contributions presented in the study are included in the article/Supplementary material, further inquiries can be directed to the corresponding author.

## Author contributions

CD: Conceptualization, Methodology, Project administration, Supervision, Writing – original draft, Writing – review & editing. RM: Conceptualization, Methodology, Project administration, Supervision, Writing – original draft, Writing – review & editing.
