# Supplementary material for: The impact of three SMN2 gene copies on clinical characteristics and effect of disease-modifying treatment in patients with spinal muscular atrophy: a systematic literature review
Source: Front Neurol. 2024 Feb 29;15:1308296. doi: 10.3389/fneur.2024.1308296 (PMC10937544; doi:10.3389/fneur.2024.1308296)
Supplement: Supplementary file 1 [file Data_Sheet_1.docx]

Supplementary Material

# Supplementary Methods

## PubMed search strategy

| #1 | (atrophy, spinal muscular[MeSH Terms]) OR (“muscular atrophy, spinal”[MeSH Terms]) |
| --- | --- |
| #2 | Spinal muscular atrophy |
| #3 | (“muscular atrophies”[Text Word] OR neuronopathies[Text Word] OR neuronopathy[Text Word] OR amyotrophies[Text Word] OR amyotrophy[Text Word]) AND (spinal[Text Word] OR spine[Text Word]) |
| #4 | #1 OR #2 OR #3 |
| #5 | (“survival of motor neuron 1 protein”[MeSH Terms]) OR (“survival or motor neuron 2 protein”[MeSH Terms]) |
| #6 | Survival motor neuron[Text Word] OR “Survival-of-motor-neuron” [Text Word] OR “Survival of motor neuron” [Text Word] OR SMN1[Text Word] or SMN2[Text Word] |
| #7 | #5 OR #6 |
| #8 | #4 AND #7 |
| #9 | “three copies”[Text Word] OR “3 copies”[Text Word] OR “four copies”[Text Word] OR “4 copies”[Text Word] OR “4 SMN2 copies”[Text Word] “four SMN2 copies”[Text Word] OR “four SMN1 copies”[Text Word] OR “4 SMN1 copies”[Text Word] OR “1-4 SMN1 gene copies”[Text Word] OR “1-4 SMN2 gene copies”[Text Word] OR “three SMN2 copies”[Text Word] OR “3 SMN2 copies”[Text Word] OR “three SMN1 copies”[Text Word] OR “3 SMN1 copies”[Text Word] OR “multiple SMN2 copies”[Text Word] OR “multiple SMN1 copies”[Text Word] |
| #10 | #8 AND #9 |
| #11 | “DNA Copy Number Variations”[Mesh] OR “Gene Duplication”[MAJR] |
| #12 | (“copy number”[Title/Abstract] OR “copy numbers”[Title/Abstract]) OR (“DNA copy number”[Other Term]) |
| #13 | #8 AND (#11 OR #12) |
| #14 | #10 OR #13 |
| #15 | #10 OR #13; Filters: English |

## Medline search strategy

| #1 | exp “survival of motor neuron 1 protein”/ |
| --- | --- |
| #2 | exp “survival of motor neuron 2 protein”/ |
| #3 | (“survival motor neuron” or “Survival-of-motor-neuron” or “Survival of Motor Neuron” or “SMN1” or SMN2”).mp |
| #4 | 1 or 2 or 3 |
| #5 | exp smn complex proteins/ |
| #6 | (“SMN protein” or “SMN gene”).mp. or “SMN”.ti,ab. |
| #7 | exp Muscular Atrophy, Spinal/ |
| #8 | (“muscular atrophy” or “muscular atrophies” or neuronopathies or neuronopathy or “spinal amytrophies” or “spinal amyotrophy”.mp. or SMA.ti. |
| #9 | “spinal muscular atrophy”.kw. |
| #10 | (5 or 6) and (7 or 8 or 9) |
| #11 | ((three or “3”) adj4 (copies or copy).ti,ab |
| #12 | 10 and 11 |
| #13 | 4 and 11 |
| #14 | 12 or 13 |
| #15 | *”DNA Copy Number Variations”/ |
| #16 | (“copy number” adj2 (changes or polymorphism* or varia*)).ti,ab |
| #17 | “dna copy number”.kw. |
| #18 | (“copy number” or “copy numbers”).ti,ab |
| #19 | 15 or 16 or 17 or 18 |
| #20 | 7 or 8 or 9 |
| #21 | 19 and 20 |
| #22 | 11 and 21 |
| #23 | 14 or 22 |
| #24 | limit 23 to English language |

## Cochrane database search strategy

| #1 | MeSH Descriptor: [Muscular Atrophy, Spinal] explode all trees |
| --- | --- |
| #2 | “muscular atrophy” OR “muscular atrophies” OR neuronopathies OR neuronopathy OR “spinal amyotrophies” OR “spinal amyotrophy” |
| #3 | Spine OR spinal |
| #4 | #2 AND #3 |
| #5 | spinal muscular atrophy |
| #6 | #1 OR #4 OR #5 |
| #7 | MeSH Descriptor: [Survival or Motor Neuron 1 Protein] explode all trees |
| #8 | MeSH Descriptor: [Survival or Motor Neuron 2 Protein] explode all trees |
| #9 | “survival motor neuron” OR “Survival-of-motor-neuron” OR “Survival or motor neuron” OR “SMN1” or “SMN2” |
| #10 | #7 OR #8 OR #9 |
| #11 | #6 AND #10 |
| #12 | “three copies” OR “3 copies” OR “four copies” OR “4 copies” OR “four SMN2 copies” OR “4 SMN2 copies” OR “three SMN2 copies” OR ‘3 SMN2 copies” or “multiple SMN2 copies” OR “increased SMN2 copies” |
| #13 | #11 and #12 |
| #14 | MeSH Descriptor: [DNA Copy Number Variations] explode all trees |
| #15 | MeSH Descriptor: [Gene Duplication] explode all trees |
| #16 | #11 AND (#14 OR #15) |
| #17 | #13 OR #16 |

## Web of Science search strategy

| #1 | ((TS=(“muscular atrophy” OR “muscular atrophies” OR neuronopathies OR neuronopathy OR “spinal amyotrophies” OR spinal amyotrophy”)) OR ALL-(“spinal muscular atrophy”)) OR TI=(SMA) |
| --- | --- |
| #2 | (TS=(“survival of motor neuron” OR “Survival-of-motor-neuron” OR “SMN1” OR “SMN2” OR “SMN protein” OR “SMN gene”)) OR TI=(SMN) |
| #3 | #1 AND #2 |
| #4 | (TI=three NEAR/4 copies)) OR TI=(3 NEAR/4 copies) |
| #5 | (AB=three NEAR/4 copies)) OR AB=(3 NEAR/4 copies) |
| #6 | (((TI=four NEAR/4 copies)) OR TI=(4 NEAR/4 copies) OR AB=(four NEAR/4 copies)) OR AB=(4 NEAR/4 copies) |
| #7 | #4 OR #5 OR #6 |
| #8 | #3 AND #7 |

# Supplementary Tables

**Supplementary Table 1.** Phenotypic subtypes of spinal muscular atrophy (1).

| **Type** | **Age of sign/symptom onset** | **Life span (untreated)** | **Motor milestones** | **Other findings** |
| --- | --- | --- | --- | --- |
| **SMA 0** | Prenatal | A few weeks, <6 months | None achieved | - Severe neonatal hypotonia - Severe weakness - Areflexia - Respiratory failure at birth - Facial diplegia - Reduced foetal movements - Atrial septal defects - Arthrogryposis |
| **SMA I** | <6 months | Median survival 8–10 months | Some head control, can only sit with support | - Loss of head control - Mild joint contractures - Normal or minimal facial weakness - Variable suck and swallow difficulties |
| **SMA II** | 6–18 months | 70% alive at 25 years | Independent sitting when placed | - Development delay with loss of motor skills - Reduced or absent deep tendon reflexes - Proximal muscle weakness - Postural tremor of fingers |
| **SMA III** | >18 months | Normal | Independent ambulation | - Proximal muscle weakness (difficulty with stairs, running) - Loss of motor skills - Fatigue - Postural tremor of fingers - Loss of patellar reflexes |
| **SMA IV** | Adulthood | Normal | Normal | - Fatigue - Proximal muscle weakness |

SMA, spinal muscular atrophy.

**Supplementary Table 2.** Studies evaluating the impact of three *SMN2* copies on disease characteristics/clinical phenotype.

| **Author, yr** | **Study design** | **Country** | **SMA type** | **No. with 3 *SMN2* copies/Total *n*** | **Assessment of factor related to *SMN2* copy number** | **Bias assessment tool used (star rating for NOS^a^)** |
| --- | --- | --- | --- | --- | --- | --- |
| Harada et al. 2002 (2) | Prospective, case control | Japan | I (*n*=11), II (*n*=14), III (*n*=2) | 18/27 | SMA type | AXIS  NOS case control (★★★★★) |
| Mailman et al. 2002 (3) | Prospective, observational | NR | I (*n*=52) or III (*n*=90) | 72/142 | SMA type | AXIS |
| Swoboda et al. 2005 (4) | Prospective, observational | USA | I (*n*=26), II (*n*=43), III (*n*=14) | 41/81 | Muscle electrophysiology (MUNE, CMAP) | NOS cohort (★★★★) |
| Cuscó et al. 2006 (5) | Prospective, observational | Spain | I (*n*=16), II (*n*=14), III (*n*=15) | 19/45 | SMA type | AXIS |
| Wirth et al. 2006 (6) | Prospective, observational | Germany | IIIa (*n*=60), IIIb (*n*=51), IV (*n*=4) | 46/115 | SMA type | AXIS |
| Tiziano et al. 2007 (7) | Prospective | Italy | II (*n*=87) | 46/87 | Motor function (HFMSE) | AXIS |
| Cobben et al. 2008 (8) | Prospective, longitudinal | Netherlands | I (*n*=34) | 3/34 | Survival | NOS cohort (★★★★★) |
| Arkblad et al. 2009 (9) | Retrospective | Sweden | I (*n*=16), II (*n*=11), III (*n*=14) | 14/41 | SMA type | AXIS |
| Elsheikh et al. 2009 (10) | Post hoc analysis of clinical trial | USA | NR | 29/45 | Muscle strength, functional parameters | AXIS |
| Rudnik-Schӧneborn et al. 2009 (11) | Retrospective | Germany | I (*n*=66) | 5/66 | Prognosis | NOS cohort (★★★★★) |
| Watihayati et al. 2009 (12) | Prospective observational | Malaysia | I (*n*=14), II (*n*=20), III (*n*=8) | 16/42 | SMA type | AXIS |
| Stratigopoulos et al. 2010 (13) | Prospective observational | USA | I (*n*=23), II (*n*=37), III (*n*=28) | 59/78 | PLS3 expression | AXIS |
| Kaufmann et al. 2011 (14) | Prospective, longitudinal | USA | II (*n*=35), III (*n*=30) | 51/65 | Motor function, muscle strength, pulmonary function | NOS cohort (★★★★★★) |
| Amara et al. 2012 (15) | Prospective, observational | Tunisia | I (*n*=16), II (*n*=6), III (*n*=2), IV (*n*=2) | Exon 7: 4/26  Exon 8: 4/36 | NAIP, p44, H4F5 and occludin copy numbers | AXIS |
| Kaufmann et al. 2012 (16) | Prospective, longitudinal | USA | II (*n*=41), III (*n*=38) | 61/79 | Motor function, pulmonary function over time | NOS cohort (★★★★★★) |
| Farrar et al. 2013 (17) | Cross-sectional | Australia | I (*n*=20), II (*n*=31), IIIa (*n*=14), IIIb (*n*=5) | NR | Motor function | NOS cohort (★★★★★★) |
| Sifi et al. 2013 (18) | Prospective | Algeria | I (*n*=15), II (*n*=12), III (*n*=33), IV (*n*=2) | 16/65 | SMA type | AXIS |
| Tiziano et al. 2013 (19) | Post hoc assessment of RCT cohort | Italy | IIIa (*n*=15) or IIIb (*n*=30) | 13/45 | Age, functional parameters, muscle strength, dyspnoea severity, ambulation, respiratory function | AXIS |
| Finkel et al. 2014 (20) | Prospective, longitudinal | USA | I (*n*=34), II (*n*=45) | 52/79 | Prognosis | NOS cohort (★★★★★★★) |
| Yamamoto et al. 2014 (21) | Prospective, observational | Japan | I (*n*=48), II (*n*=35), III (*n*=19), IV (*n*=4) | 58/106 | SMA type | AXIS |
| Yanyan et al. 2014 (22) | Cohort and case-control | China | I (*n*=19), II (*n*=21), III (*n*=25) | 50/65 | PLS3 expression | NOS case-control (★★★★★★★) |
| Brkušanin et al. 2015 (23) | Prospective, observational | Serbia | I (*n*=23), II (*n*=37), III (*n*=39) | 41/99 | SMA type | AXIS |
| Qu et al. 2015 (24) | Prospective, observational | China | I (*n*=106), II (*n*=101), III (*n*=25) | 153/232 | Age at onset, mortality | AXIS |
| Zarkov et al. 2015 (25) | Retrospective | Serbia | II (*n*=11), III (*n*=17), IV (*n*=8) | 20/36 | SMA type, gender, motor milestones, current motor performance, presence of spine deformities and limb contractures, age at loss of ambulation | AXIS |
| Medrano et al. 2016 (26) | Retrospective-prospective | Argentina | I (*n*=56), II (*n*=58), III (*n*=30) | 79/144 | SMA type | AXIS |
| Ar Rochmah et al. 2017 (27) | NR | Japan | I (*n*=61), II (*n*=37), III (n=14) | 68 ^b^/122 | Walking ability | AXIS |
| Kaneko et al. 2017 (28) | Prospective, observational | Japan | I (*n*=47), II (*n*=42), III (*n*=23) | Exon 7: 35/66  Exon 8: 31/66 | SMA type, clinical severity | AXIS |
| Calucho et al. 2018 (29) | NR | Spain | I (*n*=272), II (*n*=186), III (*n*=167) | 286/625 | SMA type | AXIS |
| De Sanctis et al. 2018 (30) | Retrospective | Italy | I (*n*=20) | 3/15 ^c^ | Phenotype and progression | NOS cohort (★★★★★★★) |
| Alves et al. 2020 (31) | Retrospective | USA | I (*n*=8), II (*n*=21), III (*n*=35), IV (*n*=1), presymptomatic (*n*=9) | 37/74 | SMN protein levels | NOS cohort (★★★★★★) |
| Coratti et al. 2020 (32) | Retrospective | Multinational | II (*n*=243) | 175/243 | Change in HFMSE over time | NOS cohort (★★★★★★★) |
| Coratti et al. 2020 (33) | Retrospective | Multinational | IIIa (*n*=136), IIIb (*n*=46) | 64/182 | Change in HFMSE over time | NOS cohort (★★★★★★★) |
| Hryshchenko et al. 2020 (34) | Retrospective | Ukraine | 0 (*n*=28), I (*n*=59), II (*n*=49), III (*n*=34) | 29/170 | SMA type | AXIS |
| Mendonça et al. 2020 (35) | Retrospective | Brazil | I (*n*=109), II (*n*=153), III (*n*=183), IV (*n*=9) | NR | SMA type | AXIS |
| Townsend et al. 2020 (36) | Retrospective | USA | I (*n*=152) or II (*n*=209) | 163/361 | Consistent stander use | NOS cohort (★★★★★★★) |
| Wadman et al. 2020 (37) | Prospective, observational | Netherlands | I (*n*=59), II (*n*=120), III (*n*=98), IV (*n*=9) | 165/286 | SMA type | AXIS |
| Zhang et al. 2020 (38) | Prospective, cohort | China | I (*n*=13), II (*n*=16), III (*n*=11) | 25/40 | Age at onset, survival | AXIS |
| Lusakowska et al. 2021 (39) | Prospective | Poland | I (*n*=140), II (*n*=182), III (*n*=344), IV (*n*=6) | 414/672 | Age at onset, ambulatory function over time (in SMA III patients only) | NOS cohort (★★★★★) |
| Ou et al. 2021 (40) | Retrospective | Taiwan | I (*n*=111) | 12/111 ^d^ | Prognosis | NOS cohort (★★★★★★★) |
| Wijaya et al. 2021 (41) | Case-control | Japan | *SMN1* deletion: I (*n*=83), II (*n*=65), III (*n*=41), IV (*n*=11)  Intragenic SMN1 mutation: I (*n*=10), II (*n*=1), III (*n*=2) | Intragenic *SMN1* mutation: 4/13 | SMA type | NOS case control (★★★★★★★) |
| Hanna et al. 2022 (42) | Retrospective | USA | I (*n*=33), II (*n*=39) | 19/45 | Hip pain | NOS cohort (★★★★★★★) |
| Krosschell et al. 2022 (43) | Longitudinal observational | USA | IIIa (*n*=44), IIIb (*n*=11), NC (*n*=1) | 25/56 | Ambulatory function (10MWT) | NOS cohort (★★★★★★) |
| Maggi et al. 2022 (44) | Retrospective, cross-sectional | Italy | II (*n*=21), III (*n*=141), IV (*n*=3) | 56/165 | Age at onset, functional parameters, SMA type, gender, sit/walk status, respiratory function | AXIS  NOS cohort (★★★★★★★) |
| Pane et al. 2022 (45) | Prospective, observational | Italy | Infants identified by neonatal screening; 0 (*n*=1), I (*n*=5), NC (*n*=11) | 3/17 | Neurological function (HNNE) | AXIS |

^a^Studies can receive between 1 and 9 stars (0–4 for Selection, 1 or 2 for Comparability, and 0–3 for Exposure (case control) or Outcomes (cohort).

^b^These 68 patients had 3 or 4 *SMN2* copies; the article compared patients with 1–2 copies vs 3–4 copies and did not state how many had 3 *SMN2* copies.

^c^*SMN2* copy number were available for 15/20 patients.

^d^*SMN2* copy number was unknown in 79 patients in this study; among those with known *SMN2* copy status, 12/32 had 3 copies.

10MWT, 10-minute walk test; AXIS, Appraisal tool for Cross-sectional Studies; CMAP, compound muscle action potential; HFMSE, Hammersmith Functional Motor Scale Expanded; HNNE, Hammersmith Neonatal and Infant Neurological Examination; MUNE, motor units innervating a distal muscle group; NC, not classified (untreated presymptomatic patients); NOS, Newcastle-Ottawa Scale; NR, not reported; RCT, randomized controlled trial; SMA, spinal muscular atrophy; SMN, survival motor neuron; USA, United States of America; yr, year.

**Supplementary Table 3.** Excluded studies on *SMN2* copies and disease characteristics and reasons for exclusion.

| **Author, yr** | **Study design** | **Country** | **SMA type** | **No. with 3 *SMN2* copies/Total *n*** | **Reason for exclusion** |
| --- | --- | --- | --- | --- | --- |
| Taylor et al. 1998 (46) | Case-control | UK | I (*n*=97), II (*n*=38), III (*n*=8) | NR | Study compared median copy number between groups with different types of SMA, but did not compare characteristics between patients with 3 *SMN2* copies and ≤2 copies |
| Kesari et al. 2005 (47) | Case-control | India | I (*n*=17), II (*n*=7), III (*n*=23), IV (*n*=3) | NR | No formal comparison undertaken between patients with 3 *SMN2* copies and ≤2 copies |
| Rudnik-Schӧneborn et al. 2008 (48) | Retrospective | Germany | I (*n*=66) | 5/66 | Focus on patients with 1 *SMN2* copy; no comparison made between patients with 3 *SMN2* copies and ≤2 copies |
| Petit et al. 2011 (49) | Retrospective | France | I (*n*=45), II (*n*=23), III (*n*=33), NC (*n*=2) | NR/103 | No formal comparison undertaken between patients with 3 *SMN2* copies or 2–4 copies |
| Carson et al. 2018 (50) | Retrospective | USA | Children with Amish (*n*=14) and Mennonite (*n*=42) haplotypes | 9/42 ^a^ | Focus was on comparing haplotypes rather than *SMN2* copy number, but 3 *SMN2* copies were associated with better survival than 2 copies in the Mennonite haplotypes |
| Alvarez et al. 2019 (51) | Prospective, observational | Chile | II (*n*=13), II (*n*=36), III (*n*=33) | 38/92 | No analysis of relationship between disease characteristics and *SMN2* copy number |
| Belter et al. 2021 (52) | Prospective, survey | USA | 2017: I (*n*=213), II (*n*=270), III (*n*=187)  2018: I (*n*=268), II (*n*=290), III (*n*=173) | 2017: 119/670  2018: 164/731 | Survey data; high number of missing datapoints for *SMN2* copy number; no comparison of clinical characteristics or outcomes between patients with 3 *SMN2* copies and ≤2 copies |
| Bowen et al. 2021 (53) | Prospective, observational | USA | NR | NR/771 | No formal comparison undertaken between patients with 3 *SMN2* copies and other copy number; however, *SMN2* copy number was inversely correlated with number of symptoms present and positively correlated with age at genetic testing |
| Souza et al. 2021 (54) | Cross-sectional | Brazil | IV (*n*=20) | 5/20 | No analysis of the impact of *SMN2* copy number on clinical characteristics |

^a^All had Mennonite haplotype M1a/M2; all Amish participants had 2 *SMN* copies.

NR, not reported; SMA, spinal muscular atrophy; SMN, survival motor neuron; UK, United Kingdom; USA, United States of America; yr, year.

**Supplementary Table 4.** Risk of bias in the cross-sectional studies examining the effects of three *SMN2* copies on clinical parameters, using the AXIS tool (55).

| **Category** | **Aim** | **Methods** | | | | | | | | | | **Results** | | | | | **Discussion** | | **Funding** | **Ethics** |
| --- | --- | --- | --- | --- | --- | --- | --- | --- | --- | --- | --- | --- | --- | --- | --- | --- | --- | --- | --- | --- |
| **Question** | **1** | **2** | **3** | **4** | **5** | **6** | **7** | **8** | **9** | **10** | **11** | **12** | **13** | **14** | **15** | **16** | **17** | **18** | **19** | **20** |
| Harada et al. 2002 (2) | Yes | Yes | No | UC | Yes | Yes | NR | Yes | Yes | Yes | Yes | Yes | NR | NR | No^a^ | Yes | Yes | No | No | Yes |
| Mailman et al. 2002 (3) | Yes | Yes | No | Yes | No | Yes | NR | Yes | Yes | No | No | Yes | NR | NR | Yes | Yes | Yes | No | No | No |
| Cuscó et al. 2006 (5) | No | Yes | No | Yes | UC | Yes | NR | Yes | Yes | No | No | Yes | No | NR | Yes | Yes | Yes | No | No | Yes |
| Wirth et al. 2006 (6) | Yes | Yes | No | Yes | UC | UC | NR | Yes | Yes | No | No | Yes | NR | NR | Yes | Yes | Yes | No | No | Yes |
| Tiziano et al. 2007 (7) | Yes | Yes | No | Yes | Yes | Yes | NR | Yes | Yes | Yes | Yes | Yes | NR | NR | Yes | Yes | Yes | No | No | Yes |
| Arkblad et al. 2009 (9) | Yes | Yes | No | Yes | Yes | Yes | NR | Yes | Yes | Partial | Partial | Yes | NR | NR | Yes | Yes | Yes | No | No | Yes |
| Elsheikh et al. 2009 (10) | Yes | Yes | No | Yes | UC | Yes | NR | Yes | Yes | Yes | Yes | Yes | NR | NR | Yes | Yes | Yes | Yes | No | UC |
| Watihayati et al. 2009 (12) | Yes | Yes | No | Yes | UC | Yes | NR | Yes | Yes | Yes | Yes | Yes | NR | NR | Yes | Yes | Yes | No | No | No |
| Stratigopoulos et al. 2010 (13) | Yes | Yes | No | Yes | Yes | Yes | NR | Yes | Yes | Yes | Yes | Yes | No | NR | Yes | Yes | Yes | No | No | No |
| Amara et al. 2012 (15) | Yes | Yes | No | Yes | Yes | Yes | NR | Yes | Yes | Yes | Yes | Yes | NR | NR | Yes | Yes | Yes | No | UC | Yes |
| Sifi et al. 2013 (18) | Yes | Yes | No | Yes | Yes | Yes | NR | Yes | Yes | No | No | Yes | No | NR | Yes | Yes | Yes | No | No | Yes |
| Tiziano et al. 2013 (19) | Yes | Yes | No | Yes | UC | UC | NR | Yes | Yes | Yes | Yes | Yes | NR | NR | Yes | Yes | Yes | No | No | Yes |
| Yamamoto et al. 2014 (21) | Yes | Yes | No | Yes | Yes | Yes | NR | Yes | Yes | Yes | Yes | Yes | NR | NR | Yes | Yes | Yes | No | No | Partial |
| Brkušanin et al. 2015 (23) | Yes | Yes | No | Yes | Yes | Yes | NR | Yes | Yes | Yes | Yes | Yes | NR | NR | Yes | Yes | Yes | No | No | Yes |
| Qu et al. 2015 (24) | Yes | Yes | No | Yes | Yes | Yes | NR | Yes | Yes | Yes | Yes | Yes | NR | NR | Yes | Yes | Yes | No | No | Yes |
| Zarkov et al. 2015 (25) | Yes | Yes | No | Yes | Yes | Yes | NR | Yes | Yes | Yes | Yes | Yes | NR | NR | Yes | Yes | Yes | No | No | Yes |
| Medrano et al. 2016 (26) | No | Yes | No | Yes | Yes | Yes | NR | Yes | Yes | Yes | No | Yes | No | NR | Yes | Yes | Yes | No | No | Partial |
| Ar Rochmah et al. 2017 (27) | Yes | Yes | No | Yes | Yes | Yes | NR | Yes | Partial | Yes | No | Yes | No | NR | Yes | Yes | Yes | Yes | No | Yes |
| Kaneko et al. 2017 (28) | Yes | Yes | No | Yes | Yes | Yes | NR | Yes | Yes | Yes | Yes | Yes | No | NR | Yes | Yes | Yes | No | No | Yes |
| Calucho et al. 2018 (29) | Yes | Yes | No | Yes | Yes | Yes | NR | Yes | Yes | Yes | Yes | Yes | NR | NR | Yes | Yes | Yes | No | No | No |
| Hryshchenko et al. 2020 (34) | Yes | Yes | No | No | Yes | Yes | NR | Partial | Yes | Yes | No | Yes | NR | NR | Yes | Yes | Yes | No | No | Yes |
| Mendonça et al. 2020 (35) | Yes | Yes | No | Yes | Yes | Yes | NR | Yes | Yes | Yes | Yes | Yes | NR | NR | Yes | Yes | Yes | Yes | No | Yes |
| Wadman et al. 2020 (37) | Yes | Yes | No | Yes | Yes | Yes | NR | Yes | Yes | Yes | Yes | Yes | NR | NR | Yes | Yes | Yes | No | No | Yes |
| Zhang et al. 2020 (38) | No | Yes | No | Yes | Yes | Yes | NR | Yes | Yes | Yes | Yes | Yes | NR | NR | Yes | Yes | Yes | Yes | UC | Yes |
| Maggi et al. 2022 (44) | Yes | Yes | No | Yes | Yes | Yes | NR | Yes | Yes | Yes | Yes | Yes | NR | NR | Yes | Yes | Yes | Yes | No | Yes |
| Pane et al. 2022 (45) | Yes | Yes | No | Yes | Yes | Yes | NR | Yes | Yes | Yes | Yes | Yes | NR | NR | Yes | Yes | Yes | No | No | Yes |

AXIS Tool questions are:

1. Were the aims/objectives of the study clear?
2. Was the study design appropriate for the stated aim(s)?
3. Was the sample size justified?
4. Was the target/reference population clearly defined? (Is it clear who the research was about?)
5. Was the sample frame taken from an appropriate population base so that it closely represented the target/reference population under investigation?
6. Was the selection process likely to select subjects/participants that were representative of the target/reference population under investigation?
7. Were measures undertaken to address and categorise non-responders?
8. Were the risk factor and outcome variables measured appropriate to the aims of the study?
9. Were the risk factor and outcome variables measured correctly using instruments/measurements that had been trialled, piloted or published previously?
10. Is it clear what was used to determined statistical significance and/or precision estimates? (e.g. p-values, confidence intervals)
11. Were the methods (including statistical methods) sufficiently described to enable them to be repeated?
12. Were the basic data adequately described?
13. Does the response rate raise concerns about non-response bias?
14. If appropriate, was information about non-responders described?
15. Were the results internally consistent?
16. Were the results presented for all the analyses described in the methods?
17. Were the authors' discussions and conclusions justified by the results?
18. Were the limitations of the study discussed?
19. Were there any funding sources or conflicts of interest that may affect the authors’ interpretation of the results?
20. Was ethical approval or consent of participants attained?

AXIS, Appraisal tool for Cross-sectional Studies; NR, not relevant; UC, unclear.

**Supplementary Table 5.** Risk of bias in the cohort or case-control studies examining the effects of three *SMN2* copies on clinical parameters, using the Newcastle-Ottawa Scale (NOS) (56).

| **NOS cohort study domains** | **Selection** | | | | **Comparability** | **Outcomes** | | |
| --- | --- | --- | --- | --- | --- | --- | --- | --- |
|  | **Representativeness of exposed cohort** | **Selection of non-exposed cohort** | **Ascertainment of exposure** | **Demonstration that outcome of interest not present at start of study** | **Comparability of cohorts based on design** | **Assessment of outcome** | **Follow-up duration adequate?** | **Adequacy of follow-up** |
| Swoboda et al. 2005 (4) | No description | NR | ★ Secure record | ★ Demonstration of change from baseline | NR | Not blinded | ★ Up to 60 months | Follow up rate <75% |
| Cobben et al. 2008 (8) | ★ Yes | NR | ★ Secure record | ★ Survival from birth | NR | ★ Medical records | ★ 3 years | Follow up rate <75% |
| Rudnick-Schöneborn et al. 2009 (11) | ★ Yes | NR | ★ Secure record | ★ Survival from birth | NR | ★ Medical records | ★Yes ^a^ | Only 38% of potential patients agreed to participate |
| Kaufmann et al. 2011 (14) | ★ Yes | NR | ★ Secure record | ★ Change from baseline | NR | ★ Consistent assessment (not blind) | ★ 1 year | ★ 4% lost to follow-up |
| Kaufmann et al. 2012 (16) | ★ Yes | NR | ★ Secure record | ★ Change from baseline | NR | ★ Consistent assessment (not blind) | ★ 25 months | ★ 14% lost to follow-up; reasons unlikely to introduce bias |
| Farrar et al. 2013 (17) | ★ Yes | NR | ★ Secure record | ★ Endpoints were mortality and clinical progression | NR | ★ Medical records | ★ Up to 25 years | ★ All patients accounted for |
| Finkel et al. 2014 (20) | ★ Somewhat | NR | ★ Secure record | ★ Endpoints were mortality and clinical progression | ★ Controlled for SMA subtype and subtype-time interaction | ★ Predefined assessment criteria | ★ 12 months | ★ Patients lost to follow-up unlikely to introduce bias |
| De Sanctis et al. 2018 (30) | ★ Yes | NR | ★ Secure record | ★ Change from baseline | ★ Controlled for *SMN2* copy number | ★ Predefined assessment criteria and trained evaluators | ★ ≥48 months | ★ All patients accounted for |
| Alves et al. 2020 (31) | ★ Somewhat | NR | ★ Secure record | ★ Change from baseline | ★ Controlled for age, sex and treatment status | ★ Record linkage | No (highly variable) | ★ All patients accounted for |
| Coratti et al. 2020 (32) | ★ Yes | NR | ★ Secure record | ★ Change from baseline | ★ Compared by age group and *SMN2* copy number | ★ Predefined assessment criteria and trained evaluators | ★ 12 months | ★ All patients accounted for |
| Coratti et al. 2020 (33) | ★ Yes | NR | ★ Secure record | ★ Change from baseline | ★ Controlled for SMA type, *SMN2* copy number, ambulatory status and sex | ★ Predefined assessment criteria and trained evaluators | ★ Median 3 years | ★ All patients accounted for |
| Townsend et al. 2020 (36) | ★ Yes | NR | ★ Secure record | ★ Stander use | ★ Controlled for SMA type, *SMN2* copy number, respiratory support, motor performance and motor control | ★ Record linkage | ★ Between 1 and 65 visits (mean 10) | ★ <10% lost to follow-up |
| Lusakowska et al. 2021 (39) | ★ Yes | NR | Written self-report | ★ Endpoints were mortality and loss of ambulation | ★ Controlled for *SMN2* copy number, sex and disease duration | Self-report | ★Yes ^a^ | ★ All patients accounted for |
| Ou et al. 2021 (40) | ★ Yes | NR | ★ Secure record | ★ Endpoints were mortality and clinical progression | ★ Controlled for *SMN2* copy number | ★ Record linkage | ★ Up to 36 years | ★ All patients accounted for |
| Hanna et al. 2022 (42) | ★ Yes | NR | ★ Secure record | ★ Endpoints were hip pain and radiographic status | ★ Controlled for SMA type | ★ Record linkage | ★ Mean 10.4 years | ★ All patients accounted for |
| Krosschell et al. 2022 (43) | ★ Yes | NR | ★ Secure record | ★ Change from baseline in 10MWT | ★ Controlled for age, BMI, SMA type, SMN2 copy number and VPA treatment | ★ Predefined assessment criteria and trained evaluators | No | ★ All patients accounted for |
| Maggi et al. 2022 (44) | ★ Yes | NR | ★ Secure record | ★ Baseline was date of diagnosis | ★ Controlled for age, sex, and *SMN2* copy number | ★ Predefined assessment criteria and trained evaluators | ★ Yes ^a^ | ★ All patients accounted for |
| **NOS case-control domains** | **Case definition adequate?** | **Representativeness of cases?** | **Selection of controls?** | **Definition of controls** | **Comparability of cases and controls** | **Ascertainment of exposure** | **Same method for cases and controls** | **Non-response rate** |
| Harada et al. 2002 (2) | ★ Yes | Potential for selection bias not stated | Community controls | ★ Healthy adult volunteers | Not very comparable and no controls | ★ Secure records | ★ Yes | ★ Same rate for both |
| Yanyan et al. 2014 (22) | ★ Yes | ★ Yes | No description | ★ Healthy children | ★ For age and gender | ★ Secure records | ★ Yes | ★ Same rate for both |
| Wijaya et al. 2021 (41) | ★ Yes | Potential for selection bias not stated | ★ SMA with homozygous *SMN1* absence | ★ Absence of *SMN1* intragenic variant | ★ Groups comparable | ★ Secure records | ★ Yes | ★ Same rate for both |

^a^Follow-up not clear, but Kaplan-Meier curves show adequate follow-up.

10MWT, 10-minute walk test; BMI, body mass index; HFMSE, Hammersmith Functional Motor Scale – Expanded; NR, not reported; SMA, spinal muscular atrophy; SMN, survival motor neuron; VPA, valproic acid.

**Supplementary Table 6.** Studies examining the effects of three *SMN2* copies on treatment effects.

| **Author, yr** | **Study design** | **Country** | **Patients** | **No. with 3 *SMN2* copies/Total *n*** | **Duration** | **Assessment parameters ^a^** | **Overall risk of bias ^b^** |
| --- | --- | --- | --- | --- | --- | --- | --- |
| **Nusinersen** | | | | | | | |
| Aragon-Gawinska et al. 2018 (57) | Prospective cohort | NR | Type I (*n*=33) aged <7 mos | 17/33 | 6 mos | HINE-2, CHOP INTEND, MFM20, MFM32, ventilatory and nutritional status | Moderate |
| Mercuri et al. 2018 (58) | Phase 3 RCT (CHERISH)^b^ | Multinational | SMA type ≥II (*n*=126) aged 2–9 yrs | 111/126 | 15 mos | HFMSE, WHO motor milestones, RULM | Some concerns |
| Pane et al. 2018 (59) | Prospective, cohort | Italy | Type I (*n*=104) aged 3 mos to 19.75 yrs | 24/104 | 6 mos | HINE, CHOP INTEND | Serious |
| De Vivo et al. 2019 (60) | Phase 2, open-label, single-arm (NURTURE) | Multinational | Presymptomatic infants aged ≤6 weeks | 10/25 | Median 2.9 yrs | Survival or respiratory intervention, HINE-2, CHOP INTEND, clinical manifestations of SMA, pNF-H levels, CMAP, achieved ambulation | Moderate |
| Pane et al. 2019 (61) | Prospective, cohort | Italy | Type I (*n*=85) aged 2 mos to 15.9 yrs | 18/85 | 12 mos | HINE, CHOP INTEND | Serious |
| Aragon-Gawinska et al. 2020 (62) | NR | Belgium, France | Type I (*n*=47) | 18/47 | 14 mos | Sitting status | Serious |
| Modrzejewska S, et al. 2021 (63) | Prospective, observational | Poland | SMA type I (*n*=26) aged 2–15 (mean 4.8) yrs | 9/26 | 18–26 mos | CHOP INTEND | Moderate |
| Vill et al. 2021 (64) | Prospective, cohort | Germany | Infants identified by newborn screening | 10/43 | NR | CHOP INTEND, HINE-2, symptoms | Serious |
| Carson et al. 2022 (65) | Prospective, cohort | USA | I (*n*=1), II (*n*=14), III (*n*=2) with complex spinal anatomy aged 2.7–31.5 yrs | 13/17 | 22–34 (median 30) mo | Weight-adjusted sum total force on dynamometry | Moderate |
| **Onasemnogene abeparvovec** | | | | | | | |
| Strauss et al. 2022 (66) | Subgroup analysis of phase III study (SPRINT) |  | Presymptomatic infants with 3 *SMN2* copies (*n*=15), aged 9–43 (median 32) days | 15/15 | 24 mos | Sit, stand, walk milestones  Adverse events | Moderate |
| **Multiple treatments ^c^** | | | | | | | |
| Lee et al. 2022 (67) | Retrospective | USA | Infants identified by newborn screening (*n*=32) | 11/32 | NR | Motor milestones | Serious |

^a^Assessment parameters in which a relationship with *SMN2* copy number was examined.

^b^Bias assessment tool was ROBINS-I for most studies, because they were mostly cohort studies, but RoB2 for phase 3 CHERISH study (58), which was randomized.

^c^Patients in this study received onasemnogene alone (*n*=23), nusinersen alone (*n*=1), risdiplam alone (*n*=1), nusinersen as a bridge to onasemnogene (*n*=5), or risdiplam after onasemnogene (*n*=2).

CHOP INTEND, Children’s Hospital of Philadelphia Infant Test of Neuromuscular Disorders; CMAP, compound muscle action potential; HFMSE, Hammersmith Functional Motor Scale Expanded; HINE, Hammersmith Infant Neurological Examination; MFM 20/32. Motor Function Measure (20 or 30 item); mo(s), month(s); MVIC, maximal voluntary isometric muscle contraction; NR, not reported; pNF-H, phosphorylated neurofilament heavy chain; RCT, randomized controlled trial; RULM, revised upper limb module; SMA-FRS, spinal muscular atrophy function rating scale; SMN, survival motor neuron; USA, United States of America; WHO World Health Organization; yr(s), year(s).

**Supplementary Table 7.** Studies excluded from the analysis of the effects of three *SMN2* copies on treatment effects.

| **Author, yr** | **Study design** | **Country** | **Patients** | **No. with 3 *SMN2* copies/Total *n*** | **Reason for exclusion** |
| --- | --- | --- | --- | --- | --- |
| **Nusinersen** | | | | | |
| Strauss et al. 2018 (68) | Prospective, cohort | USA | NR (*n*=10) aged 5.4–30.5 yrs | 8/10 | No analysis of outcomes in relation to *SMN2* copy number |
| Audic et al. 2020 (69) | Prospective, observational | France | SMA type I (*n*=34) or II (*n*=89) aged 3 mos to 16 yrs | 96/123 | No analysis of outcomes in relation to *SMN2* copy number |
| Coratti et al. 2021 (70) | Prospective, observational | Multinational | SMA type II (*n*=77) aged >30 mo (mean 7.5 yrs) | 49/77 | No comparison of outcomes in patients with different *SMN2* copy numbers |
| Elsheikh et al. 2021 (71) | Prospective, observational | USA | SMA type III (*n*=12) or IV (*n*=1) with ≥3 *SMN2* copies (*n*=13), aged 18–59 (median 36.6) yrs | 4/13 | No comparison of outcomes between patients with different *SMN2* copy numbers |
| Finkel et al. 2021 (72) | Phase 2, open-label, single-arm | Canada, USA | SMA type I (*n*=20) aged 2–7 (mean 4.6) mos | 2/20 | No analysis of treatment effect in the two patients with 3 *SMN2* copy |
| Osredkar et al. 2021 (73) | Prospective | Slovenia | SMA type I (*n*=16), II (*n*=32) or III (*n*=13) aged 0.2–18.8 (median 8.6) yrs | 37/61 | No comparison of outcomes between patients with different *SMN2* copy numbers |
| Pane et al. 2021 (74) | Prospective, cohort | Italy | SMA type I (*n*=68) aged 0.5–15.9 (mean 4.0) yrs | 17/68 | No comparison of outcomes between patients with different *SMN2* copy numbers |
| Orbach et al. 2022 (75) | Retrospective | Israel | SMA type I (*n*=22), II (*n*=17) or III (*n*=11); median age 7 yrs | NR ^a^/50 | No comparison of outcomes between patients with different *SMN2* copy numbers |
| Friese et al. 2021 (76) | Retrospective | Germany | SMA type NR (*n*=8), aged 10–39 mos | 5/8 | No comparison of outcomes between patients with different *SMN2* copy numbers |
| **Onasemnogene abeparvovec** | | | | | |
| Aharoni et al. 2022 (77) | Retrospective | Israel | SMA type I (*n*=30) or II (*n*=4), aged ≤2 yrs (mean 8.5 mos) | 8/34 | No analysis of treatment effects in relation to *SMN2* copy number (baseline comparison of AAV9 antibodies only) |
| **Valproate** | | | | | |
| Darbar et al. 2011 (78) | Prospective, open-label | Brazil | SMA type II (*n*=14) or III (*n*=8), aged 2–8 yrs | NR/33 | *SMN2* copy numbers not reported |
| **Risdiplam** | | | | | |
| Hahn et al. 2022 (79) | Prospective, observational | Germany | SMA type I (*n*=31) or II (*n*=80), aged ≥2 mos | NR/111 | *SMN2* copy numbers not reported |

^a^Patients had between 2–4 (median 3) *SMN2* copies.

AAV9, adeno-associated virus 9; mo(s), months; NR, not reported; SMA, spinal muscular atrophy; SMN, survival motor neuron; USA, United States of America; yr(s), year(s).

**Supplementary Table 8.** Bias assessments by domain in the studies examining the effects of three *SMN2* copies on treatment effects.

| **Observational/cohort studies assessed using ROBIN-I tool** | | | | | | | | | | | | | |
| --- | --- | --- | --- | --- | --- | --- | --- | --- | --- | --- | --- | --- | --- |
| **ROBINS-I domains for sources of bias** | **Confounding** | **Participant selection** | | **Classification of intervention** | | **Deviations from intended intervention** | **Missing data** | | **Outcome measurement** | | **Selection of reported results** | | **Overall** |
| Aragon-Gawinska et al. 2018 (57) | Moderate | Low | | Low | | Low | Low | | Moderate | | Moderate | | Moderate |
| Pane et al. 2018 (59) | Serious | Low | | Low | | Low | Low | | Moderate | | Moderate | | Serious |
| De Vivo et al. 2019 (60) | Moderate | Low | | Low | | Low | Low | | Low | | Moderate | | Moderate |
| Pane et al. 2019 (61) | Moderate | Low | | Low | | Low | Low | | Moderate | | Moderate | | Moderate |
| Aragon-Gawinska et al. 2020 (62) | Serious | Low | | Low | | Serious | Low | | Moderate | | Moderate | | Serious |
| Modrzejewska S, et al. 2021 (63) | Moderate | Low | | Low | | Low | Low | | Moderate | | Moderate | | Moderate |
| Vill et al. 2021 (64) | Serious | Low | | Low | | Low | Low | | Moderate | | Moderate | | Serious |
| Carson et al. 2022 (65) | Moderate | Low | | Low | | Low | Low | | Low | | Low | | Moderate |
| Strauss et al. 2022 (66) | Moderate | Low | | Low | | Low | Low | | Moderate | | Low | | Moderate |
| Lee et al. 2022 (67) | Serious | Low | | Serious | | Moderate | Low | | Moderate | | Moderate | | Serious |
| **Randomized trials assessed using the RoB-2 tool** | | | | | | | | | | | | | |
| **RoB2 domains** | **Randomization** | | **Deviations from intended interventions** | | **Missing outcome data** | | | **Outcome measurement** | | **Selection of reported results** | | **Overall** | |
| Mercuri et al. 2018 (58) | Some concerns | | Low | | Low | | | Low | | Low | | Some concerns | |

RoB-2, revised Cochrane Risk-of-Bias tool; ROBIN-I, Risk Of Bias In Non-randomized Studies – of Interventions tool.

# References

1. Prior TW, Leach ME, Finanger E. Spinal Muscular Atrophy. In: Adam MP, Everman DB, Mirzaa GM, Pagon RA, Wallace SE, Bean LJH, et al., editors. *GeneReviews®*. Seattle (WA): University of Washington, Seattle. Copyright © 1993-2022, University of Washington, Seattle. GeneReviews is a registered trademark of the University of Washington, Seattle. All rights reserved (2000).

2. Harada Y, Sutomo R, Sadewa AH, Akutsu T, Takeshima Y, Wada H, et al. Correlation between SMN2 copy number and clinical phenotype of spinal muscular atrophy: three SMN2 copies fail to rescue some patients from the disease severity. *J Neurol*. (2002) 249:1211-9. doi: 10.1007/s00415-002-0811-4.

3. Mailman MD, Heinz JW, Papp AC, Snyder PJ, Sedra MS, Wirth B, et al. Molecular analysis of spinal muscular atrophy and modification of the phenotype by SMN2. *Genet Med*. (2002) 4:20-6. doi: 10.1097/00125817-200201000-00004.

4. Swoboda KJ, Prior TW, Scott CB, McNaught TP, Wride MC, Reyna SP, et al. Natural history of denervation in SMA: relation to age, SMN2 copy number, and function. *Ann Neurol*. (2005) 57:704-12. doi: 10.1002/ana.20473.

5. Cuscó I, Barceló MJ, Rojas-García R, Illa I, Gámez J, Cervera C, et al. SMN2 copy number predicts acute or chronic spinal muscular atrophy but does not account for intrafamilial variability in siblings. *J Neurol*. (2006) 253:21-5. doi: 10.1007/s00415-005-0912-y.

6. Wirth B, Brichta L, Schrank B, Lochmüller H, Blick S, Baasner A, et al. Mildly affected patients with spinal muscular atrophy are partially protected by an increased SMN2 copy number. *Hum Genet*. (2006) 119:422-8. doi: 10.1007/s00439-006-0156-7.

7. Tiziano FD, Bertini E, Messina S, Angelozzi C, Pane M, D'Amico A, et al. The Hammersmith functional score correlates with the SMN2 copy number: a multicentric study. *Neuromuscul Disord*. (2007) 17:400-3. doi: 10.1016/j.nmd.2007.02.006.

8. Cobben JM, Lemmink HH, Snoeck I, Barth PA, van der Lee JH, de Visser M. Survival in SMA type I: a prospective analysis of 34 consecutive cases. *Neuromuscul Disord*. (2008) 18:541-4. doi: 10.1016/j.nmd.2008.05.008.

9. Arkblad E, Tulinius M, Kroksmark AK, Henricsson M, Darin N. A population-based study of genotypic and phenotypic variability in children with spinal muscular atrophy. *Acta Paediatr*. (2009) 98:865-72. doi: 10.1111/j.1651-2227.2008.01201.x.

10. Elsheikh B, Prior T, Zhang X, Miller R, Kolb SJ, Moore D, et al. An analysis of disease severity based on SMN2 copy number in adults with spinal muscular atrophy. *Muscle Nerve*. (2009) 40:652-6. doi: 10.1002/mus.21350.

11. Rudnik-Schöneborn S, Berg C, Zerres K, Betzler C, Grimm T, Eggermann T, et al. Genotype-phenotype studies in infantile spinal muscular atrophy (SMA) type I in Germany: implications for clinical trials and genetic counselling. *Clin Genet*. (2009) 76:168-78. doi: 10.1111/j.1399-0004.2009.01200.x.

12. Watihayati MS, Fatemeh H, Marini M, Atif AB, Zahiruddin WM, Sasongko TH, et al. Combination of SMN2 copy number and NAIP deletion predicts disease severity in spinal muscular atrophy. *Brain Dev*. (2009) 31:42-5. doi: 10.1016/j.braindev.2008.08.012.

13. Stratigopoulos G, Lanzano P, Deng L, Guo J, Kaufmann P, Darras B, et al. Association of plastin 3 expression with disease severity in spinal muscular atrophy only in postpubertal females. *Arch Neurol*. (2010) 67:1252-6. doi: 10.1001/archneurol.2010.239.

14. Kaufmann P, McDermott MP, Darras BT, Finkel R, Kang P, Oskoui M, et al. Observational study of spinal muscular atrophy type 2 and 3: functional outcomes over 1 year. *Arch Neurol*. (2011) 68:779-86. doi: 10.1001/archneurol.2010.373.

15. Amara A, Adala L, Ben Charfeddine I, Mamaï O, Mili A, Lazreg TB, et al. Correlation of SMN2, NAIP, p44, H4F5 and Occludin genes copy number with spinal muscular atrophy phenotype in Tunisian patients. *Eur J Paediatr Neurol*. (2012) 16:167-74. doi: 10.1016/j.ejpn.2011.07.007.

16. Kaufmann P, McDermott MP, Darras BT, Finkel RS, Sproule DM, Kang PB, et al. Prospective cohort study of spinal muscular atrophy types 2 and 3. *Neurology*. (2012) 79:1889-97. doi: 10.1212/WNL.0b013e318271f7e4.

17. Farrar MA, Vucic S, Johnston HM, du Sart D, Kiernan MC. Pathophysiological insights derived by natural history and motor function of spinal muscular atrophy. *J Pediatr*. (2013) 162:155-9. doi: 10.1016/j.jpeds.2012.05.067.

18. Sifi Y, Sifi K, Boulefkhad A, Abadi N, Bouderda Z, Cheriet R, et al. Clinical and genetic study of Algerian patients with spinal muscular atrophy. *J Neurodegener Dis*. (2013) 2013:903875. doi: 10.1155/2013/903875.

19. Tiziano FD, Lomastro R, Di Pietro L, Barbara Pasanisi M, Fiori S, Angelozzi C, et al. Clinical and molecular cross-sectional study of a cohort of adult type III spinal muscular atrophy patients: clues from a biomarker study. *Eur J Hum Genet*. (2013) 21:630-6. doi: 10.1038/ejhg.2012.233.

20. Finkel RS, McDermott MP, Kaufmann P, Darras BT, Chung WK, Sproule DM, et al. Observational study of spinal muscular atrophy type I and implications for clinical trials. *Neurology*. (2014) 83:810-7. doi: 10.1212/wnl.0000000000000741.

21. Yamamoto T, Sato H, Lai PS, Nurputra DK, Harahap NI, Morikawa S, et al. Intragenic mutations in SMN1 may contribute more significantly to clinical severity than SMN2 copy numbers in some spinal muscular atrophy (SMA) patients. *Brain Dev*. (2014) 36:914-20. doi: 10.1016/j.braindev.2013.11.009.

22. Yanyan C, Yujin Q, Jinli B, Yuwei J, Hong W, Fang S. Correlation of PLS3 expression with disease severity in children with spinal muscular atrophy. *J Hum Genet*. (2014) 59:24-7. doi: 10.1038/jhg.2013.111.

23. Brkušanin M, Kosać A, Jovanović V, Pešović J, Brajušković G, Dimitrijević N, et al. Joint effect of the SMN2 and SERF1A genes on childhood-onset types of spinal muscular atrophy in Serbian patients. *J Hum Genet*. (2015) 60:723-8. doi: 10.1038/jhg.2015.104.

24. Qu YJ, Ge XS, Bai JL, Wang LW, Cao YY, Lu YY, et al. Association of copy numbers of survival motor neuron gene 2 and neuronal apoptosis inhibitory protein gene with the natural history in a Chinese spinal muscular atrophy cohort. *J Child Neurol*. (2015) 30:429-36. doi: 10.1177/0883073814553271.

25. Zarkov M, Stojadinović A, Sekulić S, Barjaktarović I, Perić S, Keković G, et al. Association between the SMN2 gene copy number and clinical characteristics of patients with spinal muscular atrophy with homozygous deletion of exon 7 of the SMN1 gene. *Vojnosanit Pregl*. (2015) 72:859-63. doi: 10.2298/vsp140328072z.

26. Medrano S, Monges S, Gravina LP, Alías L, Mozzoni J, Aráoz HV, et al. Genotype-phenotype correlation of SMN locus genes in spinal muscular atrophy children from Argentina. *Eur J Paediatr Neurol*. (2016) 20:910-7. doi: 10.1016/j.ejpn.2016.07.017.

27. Ar Rochmah M, Shima A, Harahap NIF, Niba ETE, Morisada N, Yanagisawa S, et al. Gender effects on the clinical phenotype in Japanese patients with spinal muscular atrophy. *Kobe J Med Sci*. (2017) 63:E41-e4.

28. Kaneko K, Arakawa R, Urano M, Aoki R, Saito K. Relationships between long-term observations of motor milestones and genotype analysis results in childhood-onset Japanese spinal muscular atrophy patients. *Brain Dev*. (2017) 39:763-73. doi: 10.1016/j.braindev.2017.04.018.

29. Calucho M, Bernal S, Alías L, March F, Venceslá A, Rodríguez-Álvarez FJ, et al. Correlation between SMA type and SMN2 copy number revisited: an analysis of 625 unrelated Spanish patients and a compilation of 2834 reported cases. *Neuromuscul Disord*. (2018) 28:208-15. doi: 10.1016/j.nmd.2018.01.003.

30. De Sanctis R, Pane M, Coratti G, Palermo C, Leone D, Pera MC, et al. Clinical phenotypes and trajectories of disease progression in type 1 spinal muscular atrophy. *Neuromuscul Disord*. (2018) 28:24-8. doi: 10.1016/j.nmd.2017.09.015.

31. Alves CRR, Zhang R, Johnstone AJ, Garner R, Eichelberger EJ, Lepez S, et al. Whole blood survival motor neuron protein levels correlate with severity of denervation in spinal muscular atrophy. *Muscle Nerve*. (2020) 62:351-7. doi: 10.1002/mus.26995.

32. Coratti G, Lucibello S, Pera MC, Duong T, Muni Lofra R, Civitello M, et al. Gain and loss of abilities in type II SMA: a 12-month natural history study. *Neuromuscul Disord*. (2020) 30:765-71. doi: 10.1016/j.nmd.2020.07.004.

33. Coratti G, Messina S, Lucibello S, Pera MC, Montes J, Pasternak A, et al. Clinical variability in spinal muscular atrophy type III. *Ann Neurol*. (2020) 88:1109-17. doi: 10.1002/ana.25900.

34. Hryshchenko NV, Yurchenko AA, Karaman HS, Livshits LA. Genetic modifiers of the spinal muscular atrophy phenotype. *Cytol Genet*. (2020) 54:130-6. doi: 10.3103/s0095452720020073.

35. Mendonça RH, Matsui C, Jr., Polido GJ, Silva AMS, Kulikowski L, Torchio Dias A, et al. Intragenic variants in the SMN1 gene determine the clinical phenotype in 5q spinal muscular atrophy. *Neurol Genet*. (2020) 6:e505. doi: 10.1212/nxg.0000000000000505.

36. Townsend EL, Simeone SD, Krosschell KJ, Zhang RZ, Swoboda KJ, Project Cure SMA Investigator's Network. Stander use in spinal muscular atrophy: results from a large natural history database. *Pediatr Phys Ther*. (2020) 32:235-41. doi: 10.1097/pep.0000000000000713.

37. Wadman RI, Jansen MD, Stam M, Wijngaarde CA, Curial CAD, Medic J, et al. Intragenic and structural variation in the SMN locus and clinical variability in spinal muscular atrophy. *Brain Commun*. (2020) 2:fcaa075. doi: 10.1093/braincomms/fcaa075.

38. Zhang Y, He J, Zhang Y, Li L, Tang X, Wang L, et al. The analysis of the association between the copy numbers of survival motor neuron gene 2 and neuronal apoptosis inhibitory protein genes and the clinical phenotypes in 40 patients with spinal muscular atrophy: Observational study. *Medicine (Baltimore)*. (2020) 99:e18809. doi: 10.1097/md.0000000000018809.

39. Lusakowska A, Jedrzejowska M, Kaminska A, Janiszewska K, Grochowski P, Zimowski J, et al. Observation of the natural course of type 3 spinal muscular atrophy: data from the polish registry of spinal muscular atrophy. *Orphanet J Rare Dis*. (2021) 16:150. doi: 10.1186/s13023-021-01771-y.

40. Ou SF, Ho CS, Lee WT, Lin KL, Jones CC, Jong YJ. Natural history in spinal muscular atrophy type I in Taiwanese population: a longitudinal study. *Brain Dev*. (2021) 43:127-34. doi: 10.1016/j.braindev.2020.07.012.

41. Wijaya YOS, Ar Rohmah M, Niba ETE, Morisada N, Noguchi Y, Hidaka Y, et al. Phenotypes of SMA patients retaining SMN1 with intragenic mutation. *Brain Dev*. (2021) 43:745-58. doi: 10.1016/j.braindev.2021.03.006.

42. Hanna RB, Nahm N, Bent MA, Sund S, Patterson K, Schroth MK, et al. Hip pain in nonambulatory children with type-I or II spinal muscular atrophy. *JB JS Open Access*. (2022) 7:e22.00011. doi: 10.2106/jbjs.Oa.22.00011.

43. Krosschell KJ, Townsend EL, Kiefer M, Simeone SD, Zumpf K, Welty L, et al. Natural history of 10-meter walk/run test performance in spinal muscular atrophy: a longitudinal analysis. *Neuromuscul Disord*. (2022) 32:125-34. doi: 10.1016/j.nmd.2021.08.010.

44. Maggi L, Bello L, Bonanno S, Govoni A, Caponnetto C, Passamano L, et al. Adults with spinal muscular atrophy: a large-scale natural history study shows gender effect on disease. *J Neurol Neurosurg Psychiatry*. (2022). doi: 10.1136/jnnp-2022-329320.

45. Pane M, Donati MA, Cutrona C, De Sanctis R, Pirinu M, Coratti G, et al. Neurological assessment of newborns with spinal muscular atrophy identified through neonatal screening. *Eur J Pediatr*. (2022) 181:2821-9. doi: 10.1007/s00431-022-04470-3.

46. Taylor JE, Thomas NH, Lewis CM, Abbs SJ, Rodrigues NR, Davies KE, et al. Correlation of SMNt and SMNc gene copy number with age of onset and survival in spinal muscular atrophy. *Eur J Hum Genet*. (1998) 6:467-74. doi: 10.1038/sj.ejhg.5200210.

47. Kesari A, Idris MM, Chandak GR, Mittal B. Genotype-phenotype correlation of SMN locus genes in spinal muscular atrophy patients from India. *Exp Mol Med*. (2005) 37:147-54. doi: 10.1038/emm.2005.20.

48. Rudnik-Schöneborn S, Heller R, Berg C, Betzler C, Grimm T, Eggermann T, et al. Congenital heart disease is a feature of severe infantile spinal muscular atrophy. *J Med Genet*. (2008) 45:635-8. doi: 10.1136/jmg.2008.057950.

49. Petit F, Cuisset JM, Rouaix-Emery N, Cancés C, Sablonnière B, Bieth E, et al. Insights into genotype-phenotype correlations in spinal muscular atrophy: a retrospective study of 103 patients. *Muscle Nerve*. (2011) 43:26-30. doi: 10.1002/mus.21832.

50. Carson VJ, Puffenberger EG, Bowser LE, Brigatti KW, Young M, Korulczyk D, et al. Spinal muscular atrophy within Amish and Mennonite populations: Ancestral haplotypes and natural history. *PLoS One*. (2018) 13:e0202104. doi: 10.1371/journal.pone.0202104.

51. Alvarez K, Suarez B, Palomino MA, Hervias C, Calcagno G, Martínez-Jalilie M, et al. Observations from a nationwide vigilance program in medical care for spinal muscular atrophy patients in Chile. *Arq Neuropsiquiatr*. (2019) 77:470-7. doi: 10.1590/0004-282x20190073.

52. Belter L, Jarecki J, Reyna SP, Cruz R, Jones CC, Schroth M, et al. The Cure SMA Membership Surveys: highlights of key demographic and clinical characteristics of individuals with spinal muscular atrophy. *J Neuromuscul Dis*. (2021) 8:109-23. doi: 10.3233/jnd-200563.

53. Bowen BM, Truty R, Aradhya S, Bristow SL, Johnson BA, Morales A, et al. SMA identified: clinical and molecular findings from a sponsored testing program for spinal muscular atrophy in more than 2,000 individuals. *Front Neurol*. (2021) 12:663911. doi: 10.3389/fneur.2021.663911.

54. Souza PVS, Pinto W, Ricarte A, Badia BML, Seneor DD, Teixeira DT, et al. Clinical and radiological profile of patients with spinal muscular atrophy type 4. *Eur J Neurol*. (2021) 28:609-19. doi: 10.1111/ene.14587.

55. Downes MJ, Brennan ML, Williams HC, Dean RS. Development of a critical appraisal tool to assess the quality of cross-sectional studies (AXIS). *BMJ Open*. (2016) 6:e011458. doi: 10.1136/bmjopen-2016-011458.

56. Wells GA, Shea B, O'Connell D, Peterson J, Welch V, Losos M, et al. The Newcastle-Ottawa Scale (NOS) for assessing the quality of nonrandomised studies in meta-analyses: The Ottawa Hospital Research Institute (2021) [cited 2023 May 5]. Available from: <https://www.ohri.ca/programs/clinical_epidemiology/oxford.asp>.

57. Aragon-Gawinska K, Seferian AM, Daron A, Gargaun E, Vuillerot C, Cances C, et al. Nusinersen in patients older than 7 months with spinal muscular atrophy type 1: a cohort study. *Neurology*. (2018) 91:e1312-e8. doi: 10.1212/wnl.0000000000006281.

58. Mercuri E, Darras BT, Chiriboga CA, Day JW, Campbell C, Connolly AM, et al. Nusinersen versus sham control in later-onset spinal muscular atrophy. *N Engl J Med*. (2018) 378:625-35. doi: 10.1056/NEJMoa1710504.

59. Pane M, Palermo C, Messina S, Sansone VA, Bruno C, Catteruccia M, et al. Nusinersen in type 1 SMA infants, children and young adults: preliminary results on motor function. *Neuromuscul Disord*. (2018) 28:582-5. doi: 10.1016/j.nmd.2018.05.010.

60. De Vivo DC, Bertini E, Swoboda KJ, Hwu WL, Crawford TO, Finkel RS, et al. Nusinersen initiated in infants during the presymptomatic stage of spinal muscular atrophy: interim efficacy and safety results from the phase 2 NURTURE study. *Neuromuscul Disord*. (2019) 29:842-56. doi: 10.1016/j.nmd.2019.09.007.

61. Pane M, Coratti G, Sansone VA, Messina S, Bruno C, Catteruccia M, et al. Nusinersen in type 1 spinal muscular atrophy: Twelve-month real-world data. *Ann Neurol*. (2019) 86:443-51. doi: 10.1002/ana.25533.

62. Aragon-Gawinska K, Daron A, Ulinici A, Vanden Brande L, Seferian A, Gidaro T, et al. Sitting in patients with spinal muscular atrophy type 1 treated with nusinersen. *Dev Med Child Neurol*. (2020) 62:310-4. doi: 10.1111/dmcn.14412.

63. Modrzejewska S, Kotulska K, Kopyta I, Grędowska E, Emich-Widera E, Tomaszek K, et al. Nusinersen treatment of Spinal Muscular Atrophy Type 1 - results of expanded access programme in Poland. *Neurol Neurochir Pol*. (2021) 55:289-94. doi: 10.5603/PJNNS.a2021.0020.

64. Vill K, Schwartz O, Blaschek A, Gläser D, Nennstiel U, Wirth B, et al. Newborn screening for spinal muscular atrophy in Germany: clinical results after 2 years. *Orphanet J Rare Dis*. (2021) 16:153. doi: 10.1186/s13023-021-01783-8.

65. Carson VJ, Young M, Brigatti KW, Robinson DL, Reed RM, Sohn J, et al. Nusinersen by subcutaneous intrathecal catheter for symptomatic spinal muscular atrophy patients with complex spine anatomy. *Muscle Nerve*. (2022) 65:51-9. doi: 10.1002/mus.27425.

66. Strauss KA, Farrar MA, Muntoni F, Saito K, Mendell JR, Servais L, et al. Onasemnogene abeparvovec for presymptomatic infants with three copies of SMN2 at risk for spinal muscular atrophy: the Phase III SPR1NT trial. *Nat Med*. (2022) 28:1390-7. doi: 10.1038/s41591-022-01867-3.

67. Lee BH, Deng S, Chiriboga CA, Kay DM, Irumudomon O, Laureta E, et al. Newborn screening for spinal muscular atrophy in New York state: clinical outcomes from the first 3 years. *Neurology*. (2022) 99:e1527-37. doi: 10.1212/wnl.0000000000200986.

68. Strauss KA, Carson VJ, Brigatti KW, Young M, Robinson DL, Hendrickson C, et al. Preliminary safety and tolerability of a novel subcutaneous intrathecal catheter system for repeated outpatient dosing of nusinersen to children and adults with spinal muscular atrophy. *J Pediatr Orthop*. (2018) 38:e610-e7. doi: 10.1097/bpo.0000000000001247.

69. Audic F, de la Banda MGG, Bernoux D, Ramirez-Garcia P, Durigneux J, Barnerias C, et al. Effects of nusinersen after one year of treatment in 123 children with SMA type 1 or 2: a French real-life observational study. *Orphanet J Rare Dis*. (2020) 15:148. doi: 10.1186/s13023-020-01414-8.

70. Coratti G, Pane M, Lucibello S, Pera MC, Pasternak A, Montes J, et al. Age related treatment effect in type II spinal muscular atrophy pediatric patients treated with nusinersen. *Neuromuscul Disord*. (2021) 31:596-602. doi: 10.1016/j.nmd.2021.03.012.

71. Elsheikh B, Severyn S, Zhao S, Kline D, Linsenmayer M, Kelly K, et al. Safety, tolerability, and effect of nusinersen treatment in ambulatory adults with 5q-SMA. *Front Neurol*. (2021) 12:650535. doi: <https://dx.doi.org/10.3389/fneur.2021.650535>.

72. Finkel RS, Chiriboga CA, Vajsar J, Day JW, Montes J, De Vivo DC, et al. Treatment of infantile-onset spinal muscular atrophy with nusinersen: final report of a phase 2, open-label, multicentre, dose-escalation study. *Lancet Child Adolesc Health*. (2021) 5:491-500. doi: <https://dx.doi.org/10.1016/S2352-4642(21)00100-0>.

73. Osredkar D, Jílková M, Butenko T, Loboda T, Golli T, Fuchsová P, et al. Children and young adults with spinal muscular atrophy treated with nusinersen. *Eur J Paediatr Neurol*. (2021) 30:1-8. doi: 10.1016/j.ejpn.2020.11.004.

74. Pane M, Coratti G, Sansone VA, Messina S, Catteruccia M, Bruno C, et al. Type I SMA "new natural history": long-term data in nusinersen-treated patients. *Ann Clin Transl Neurol*. (2021) 8:548-57. doi: 10.1002/acn3.51276.

75. Orbach R, Sagi L, Sadot E, Tokatly Latzer I, Shtamler A, Zisberg T, et al. Cerebrospinal fluid characteristics of patients treated with intrathecal nusinersen for spinal muscular atrophy. *Muscle Nerve*. (2022) 66:762-6. doi: 10.1002/mus.27731.

76. Friese J, Geitmann S, Holzwarth D, Müller N, Sassen R, Baur U, et al. Safety monitoring of gene therapy for spinal muscular atrophy with onasemnogene abeparvovec -a single centre experience. *J Neuromuscul Dis*. (2021) 8:209-16. doi: 10.3233/jnd-200593.

77. Aharoni S, Bistritzer J, Levine H, Sagi L, Fattal-Valevski A, Ginzberg M, et al. Adeno-associated virus serotype 9 antibody titers in patients with SMA pre-screened for treatment with onasemnogene abeparvovec -routine care evidence. *Gene Ther*. (2023) 30:101-6. doi: 10.1038/s41434-022-00339-0.

78. Darbar IA, Plaggert PG, Resende MB, Zanoteli E, Reed UC. Evaluation of muscle strength and motor abilities in children with type II and III spinal muscle atrophy treated with valproic acid. *BMC Neurol*. (2011) 11:36. doi: 10.1186/1471-2377-11-36.

79. Hahn A, Gunther R, Ludolph A, Schwartz O, Trollmann R, Weydt P, et al. Short-term safety results from compassionate use of risdiplam in patients with spinal muscular atrophy in Germany. *Orphanet J Rare Dis*. (2022) 17:10. doi: 10.1186/s13023-022-02420-8.
